# Serum 25-hydroxyvitamin D and health-related quality of life in patients with papillary thyroid carcinoma: a prospective cohort study

**DOI:** 10.3389/fnut.2026.1782902

**Published:** 2026-03-10

**Authors:** Yan Li, Ying Wei, Zhenlong Zhao, Lili Peng, Shuqi Li, Jie Wu, Shiliang Cao, Na Yu, Wenjia Cai, Qian Wu, Song Li, Ming-an Yu

**Affiliations:** Department of Interventional Medicine, China-Japan Friendship Hospital, Beijing, China

**Keywords:** 25-hydroxyvitamin D, health-related quality of life, papillary thyroid carcinoma, vitamin D, weight gain

## Abstract

**Background:**

The incidence of papillary thyroid carcinoma (PTC) is rising worldwide. Despite its excellent prognosis, many PTC patients experience impaired Health-Related quality of life (HRQoL). Vitamin D exerts pleiotropic effects on musculoskeletal, immune, and psychological health, but its impact on HRQoL in PTC patients remains unclear.

**Methods:**

This prospective cohort study consecutively recruited patients with PTC from January 2024 to August 2025. Baseline serum 25-hydroxyvitamin D concentrations were measured before treatment. The European Organization for Research and Treatment of Cancer Quality of Life Questionnaire-Core 30 (EORTC QLQ-C30) and The Thyroid Cancer-Specific Quality of Life Questionnaire (THYCA-QoL) were applied to assess HRQoL. Associations between serum 25-hydroxyvitamin D and HRQoL domains were examined using rank-transformed general linear models, spearman correlation, multivariable linear regression and binary logistic regression. A nomogram was developed to predict the probability of weight gain in HRQoL domains and evaluated using Harrell’s C-index and bootstrap calibration.

**Results:**

Among 600 patients, 27.8% had vitamin D deficiency, 69.5% had insufficiency, and 2.7% had sufficiency. Patients with sufficient vitamin D had better physical functioning and lower social functioning scores than those with deficiency or insufficiency (*p* = 0.002). Vitamin D was positively correlated with multiple functioning domains and negatively correlated with specific symptoms (all *p* < 0.05). In linear and logistic regression, higher vitamin D levels were independently associated with better physical and role functioning and fewer symptom burdens, and reduced the odds of impaired physical functioning (OR = 0.986, *p* = 0.016) and neuromuscular and weight gain symptoms (all *p* < 0.05). The nomogram showed fair discrimination (C-index 0.628) and good calibration.

**Conclusion:**

Suboptimal vitamin D status was common among patients with PTC. Higher vitamin D levels were associated with better physical and role functioning and fewer neuromuscular, psychological, and weight gain symptoms. Assessment of vitamin D status may be considered as part of supportive care when clinically indicated.

## Introduction

The global incidence of thyroid cancer has been rising rapidly over recent decades, with a particularly marked increase in China (1, 2). Papillary thyroid carcinoma (PTC) is the most common histological subtype, accounting for the majority of thyroid malignancies (3). Although PTC generally carries a favorable prognosis, with over 90% 10-year overall survival rate (4), many patients experience persistent symptoms, such as fatigue, neuromuscular symptoms, anxiety, and depression, that may substantially impair health-related quality of life (HRQoL) (5–7). These physical and psychological burdens are well reported among thyroid cancer patients and have been shown to negatively affect HRQoL (8).

HRQoL is a key endpoint and a strong predictor of survival in medical and health research, reflecting the impact of disease and treatment on impairments and functional status. It indicates treatment success, carries prognosis value, and serves as an important factor in medical decision-making (9). Identifying factors associated with HRQoL in PTC is essential for developing comprehensive management strategies.

Vitamin D, a steroid hormone primarily known for its role in calcium and bone metabolism, also exerts pleiotropic effects on immune regulation, muscle function, and neuropsychological health (10, 11). Vitamin D deficiency is highly prevalent worldwide and has been associated with a wide range of health conditions, including osteoporosis, autoimmune diseases, and several cancers (12–15). In oncology populations, higher vitamin D status has been linked to improved HRQoL outcomes, including reduced fatigue and better physical functioning in breast cancer survivors, enhanced overall health status in colorectal cancer patients (16, 17). These findings suggest that vitamin D may play an important role in shaping patient-reported HRQoL outcomes. However, in thyroid cancer research, greater emphasis has been placed on the association between vitamin D levels and thyroid cancer risk and prognostic outcomes (18, 19), the potential impact of vitamin D on HRQoL outcomes in patients with PTC remains limited.

Against this background, we conduct a prospective cohort study to investigate the association between serum 25-hydroxyvitamin D and HRQoL in patients with PTC. Clarifying this relationship may inform routine nutritional assessment and support the development of targeted, potentially modifiable strategies to improve patient-reported outcomes in this growing population.

## Methods

### Patients

This study was a cross-sectional baseline of prospective cohort. Consecutive patients with fine-needle aspiration–confirmed PTC were recruited between January 2024 and August 2025. Serum 25-hydroxyvitamin D and HRQoL were assessed at baseline prior to definitive treatment. The parent cohort includes postoperative follow-up assessments at 3, 6, and 12 months; however, the present analysis was restricted to baseline measurements.

The study was approved by the Institutional Review Board (Approval Number: 2023-KY-250) and registered at ClinicalTrials.gov (NCT06610604). Written informed consent was obtained from all participants prior to enrollment, and all procedures were conducted in accordance with the Declaration of Helsinki. Inclusion criteria were: (a) pathological confirmation of PTC by fine-needle aspiration (FNA); (b) age between 18 and 80 years; and (c) ability to understand and complete questionnaires independently. Exclusion criteria included: (a) vitamin D supplementation within the past 3 months, (b) clinically significant renal or hepatic dysfunction, (c) concurrent malignancies or other severe comorbidities likely to affect HRQoL, and (d) psychiatric disorders.

### Data collection

Sociodemographic data, including age, sex, place of residence, and family history of cancer, were obtained from patient records. Laboratory parameters and nodule characteristics were extracted from the hospital’s electronic medical record system. Anthropometric data, including height and weight, were objectively measured by trained medical staff at hospital admission. BMI was calculated as weight (kg)/height (m^2^).

### Serum 25-hydroxyvitamin D assessment

Serum 25(OH)D was measured at baseline, 1 day before definitive treatment, and all laboratory tests were performed by the hospital’s central laboratory. Sunlight exposure season was classified as high sunlight exposure (May–October) and low sunlight exposure (November–April) based on the timing of vitamin D testing. Serum 25-hydroxyvitamin D [25(OH)D] concentrations were determined using a standardized enzyme immunoassay (EIA) kit (Immunodiagnostic Systems Limited, Boldon, United Kingdom). Vitamin D status was classified as deficiency (<25 nmol/L), insufficiency (25–75 nmol/L), and sufficiency (75–250 nmol/L), based on hospital clinical practice and supported by previous literature (20).

### HRQoL assessment

The European Organization for Research and Treatment of Cancer Quality of Life Questionnaire-Core 30 (EORTC QLQ-C30) assesses general cancer-related QoL and includes five functional scales, three symptom scales, six single-item symptoms, and a global health status scale. Scores are transformed to a 0–100 scale, with higher functional and global health scores indicating better HRQoL, and higher symptom scores indicating greater symptom burden (21).

The Thyroid Cancer-Specific Quality of Life Questionnaire (THYCA-QoL) was applied to capture thyroid-related symptoms. It comprises seven multi-item symptom scales and six single items, all scored on a 0–100 scale, with higher scores reflecting more severe thyroid-specific complaints, including self-reported weight gain (22).

HRQoL questionnaires were completed at baseline prior to treatment.

### Nomogram development and validation

The weight gain domain was selected for nomogram development because weight gain is a common survivorship concern in patients with PTC and is clinically relevant. In our multivariable analyses across HRQoL domains, the weight gain domain showed the most consistent independent association with serum vitamin D. Therefore, we conducted an exploratory nomogram analysis to provide an interpretable estimate of the probability of reporting weight-gain symptoms.

The nomogram was constructed based on the final multivariable logistic regression model for the dichotomized weight gain outcome. Discrimination was assessed using Harrell’s C-index, and the 95% confidence interval was estimated using bootstrap resampling (1,000 iterations). Calibration was evaluated using bootstrap-corrected calibration curves (1,000 resamples), with mean absolute error reported.

### Statistical analysis

Continuous variables were expressed as mean ± SD or medians with interquartile ranges (IQRs), and categorical variables as frequencies and percentages. Differences in HRQoL domains across vitamin D status groups (deficiency, insufficiency, sufficiency) were examined using rank-transformed general linear models (GLM), adjusted for age, sex, BMI, sunlight exposure season, medical insurance, family history of cancers, place of residence, and education level. *Post-hoc* pairwise comparisons were adjusted using Bonferroni correction. Spearman’s correlation was used to evaluate associations between vitamin D levels and HRQoL scores, with a heatmap visualizing these relationships.

Multivariable linear regression analyzed the impact of vitamin D levels on HRQoL scores, adjusting for age, sex, BMI, sunlight exposure season, medical insurance, family history of cancer, place of residence, and education level. Binary logistic regression further assessed the relationship between vitamin D and HRQoL status, with HRQoL scores dichotomized based on median values. Three models were fitted: Model 1 (unadjusted), Model 2 (adjusted for age and sex), and Model 3 (multivariable adjusted). Model 3 was adjusted for age, sex, BMI, sunlight exposure season, medical insurance, family history of cancer, place of residence, education level, maximum nodule diameter, and nodule focality. Nomogram-related analyses were conducted as described above.

All statistical analyses and figure construction were performed using SPSS (version 26.0) and R (4.5.1). A two-sided *p*-value <0.05 was considered statistically significant.

## Results

### Sociodemographic and clinical characteristics

**Figure 1:**
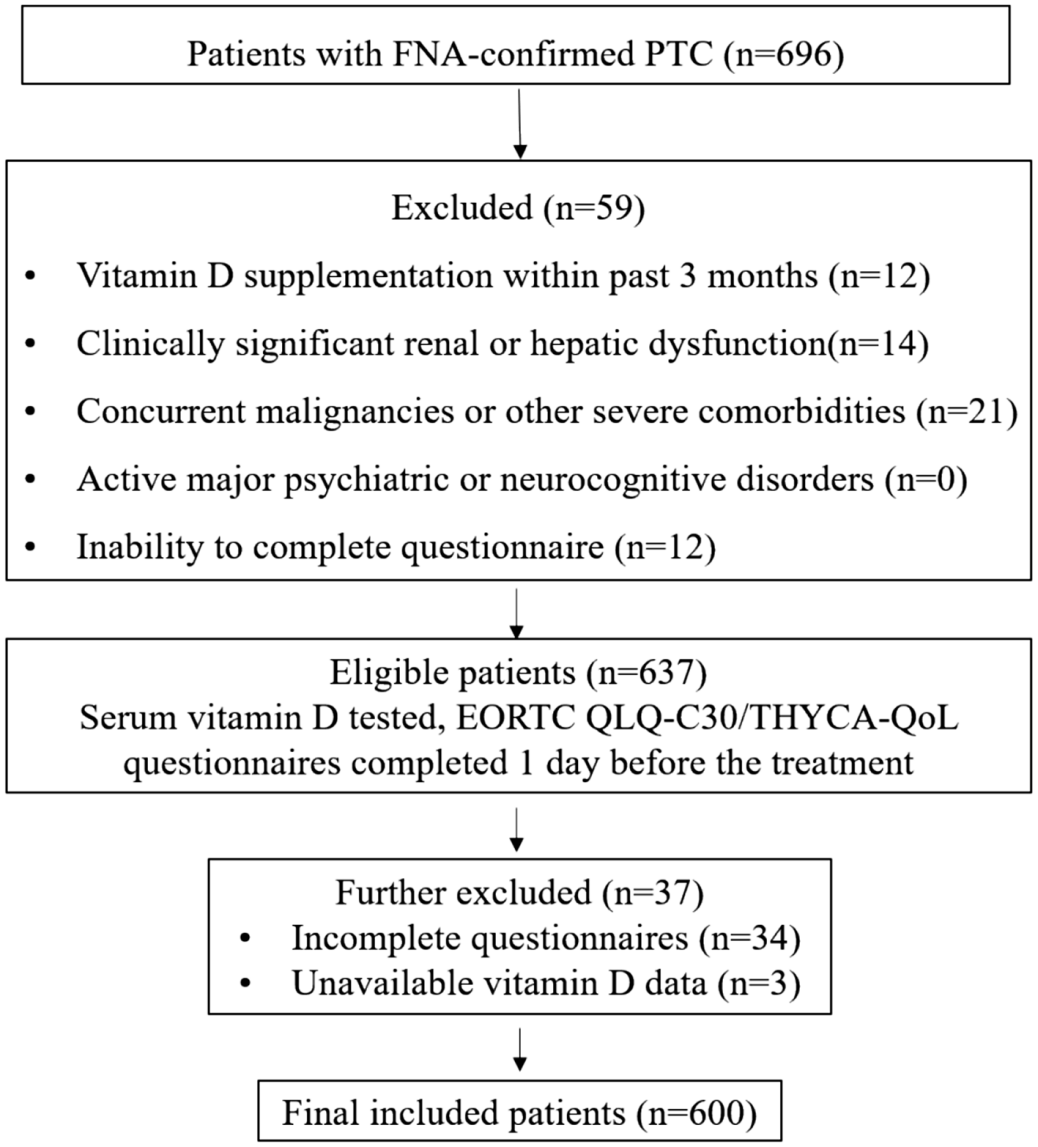
Flowchart of patient enrollment in this prospective cohort study. FNA, fine needle aspiration; PTC, papillary thyroid carcinoma.

A total of 696 patients with FNA-confirmed PTC were enrolled. After excluding 96 patients who did not meet the inclusion criteria, 600 patients were included in the final analysis (Figure 1).

Among the 600 patients, 433 were women (72.2%) and 167 were men (27.8%). The median age was 39 years, and 63.7% were <45 years. Most had a normal BMI (59.2%), lived in urban areas (87.8%), and had medical insurance (94.2%). More than half had completed college (56.7%), and 20.0% reported a family history of cancer. Unifocal disease was present in 59.0% of cases, and 40.7% had a maximum nodule diameter ≥1 cm (Table 1).

**Table 1:** Sociodemographic and clinical characteristics of patients (*n* = 600).

Variable	*N*	(%)	Median (IQRs)
Sex			
Female	433	72.2	
Male	167	27.8	
Age (years)			39.0 (33.0–49.0)
<45	382	63.7	
≥45	218	36.3	
BMI (kg/m^2^)			24.00 (21.4–26.6)
Underweight (<18.5)	25	4.2	
Normal weight (18.5–24.9)	355	59.2	
Overweight (25–29.9)	181	30.2	
Obesity class I (30–34.9)	35	5.8	
Obesity class II (35–39.9)	3	0.5	
Obesity class III (≥40)	1	0.2	
Medical insurance			
Yes	565	94.2	
No	35	5.8	
Education level			
Primary/below	17	2.8	
Middle/high school	153	25.5	
College	340	56.7	
College above	90	15.0	
Sunlight exposure season			
High sunlight	329	54.8	
Low sunlight	271	45.2	
Place of residence			
Urban	527	87.8	
Rural	73	12.2	
Family history of cancer			
Yes	120	20.0	
No	480	80.0	
Nodule focality			
Unifocal	354	59.0	
Multifocal	246	41.0	
Maximum nodule diameter (cm)			0.8 (0.6–1.2)
<1	356	59.3	
≥1	244	40.7	

Data are presented as number, percentage, and medians with IQRs. BMI was classified according to the World Health Organization (WHO) criteria.

### Vitamin D levels and status

The median serum 25(OH)D was 34.40 (23.95–45.05) nmol/L. Overall, 167 (27.8%) patients had vitamin D deficiency, 417 (69.5%) patients had insufficiency, and only 16 (2.7%) achieved sufficiency. These results indicate that the majority of patients suffered from inadequate vitamin D levels (Table 2).

**Table 2:** Serum vitamin D levels and status in patients with PTC (*n* = 600).

Variable	*N* (%)
Serum 25(OH)D (nmol/L)	34.40 (23.95–45.05)
Deficiency (<25 nmol/L)	167 (27.8)
Insufficiency (25–75 nmol/L)	417 (69.5)
Sufficiency (75–250 nmol/L)	16 (2.7)

Data are presented as number, percentage, and medians with IQRs. 25(OH)D, 25-hydroxyvitamin D.

### Comparison of HRQoL across vitamin D status (rank-transformed GLM)

After adjustment, patients with sufficient vitamin D had significantly highest estimated marginal mean physical functioning scores (415.68 ± 45.60) and lowest estimated marginal mean social functioning scores (166.91 ± 36.57), significant differences were found across different vitamin D status (*F* = 4.836, *p* = 0.008; *F* = 6.424, *p* = 0.002) compared with those with deficiency or insufficiency. No statistically significant differences were found in other domains (Table 3). Bonferroni-adjusted *post-hoc* pairwise comparisons are provided in Supplementary Table S1.

**Table 3:** Comparison of QoL scores across vitamin D status using rank-transformed GLM.

QoL domain	Deficiency (*n* = 167)	Insufficiency (*n* = 417)	Sufficiency (*n* = 16)	*F*	*p*
EORTC QLQ-C30					
Global health^+^	275.38 ± 22.39	269.97 ± 19.89	256.06 ± 46.91	0.115	0.892
Physical functioning^+^	293.72 ± 21.77	327.95 ± 19.33	415.68 ± 45.60	4.836	0.008^*^
Role functioning^+^	285.75 ± 17.76	306.46 ± 15.77	323.09 ± 37.20	1.47	0.230
Emotion functioning^+^	316.52 ± 21.90	319.91 ± 19.44	367.99 ± 45.88	0.674	0.510
Cognitive functioning^+^	306.12 ± 21.68	315.96 ± 19.25	335.55 ± 45.43	0.325	0.722
Social functioning^+^	285.51 ± 17.45	289.96 ± 15.50	166.91 ± 36.57	6.424	0.002^*^
Fatigue^++^	271.38 ± 22.03	248.72 ± 19.56	216.99 ± 46.15	1.35	0.260
Nausea/vomiting^++^	277.01 ± 18.70	291.97 ± 16.60	241.99 ± 39.17	1.38	0.251
Pain^++^	266.93 ± 19.96	274.10 ± 17.72	219.51 ± 41.82	1.03	0.358
Dyspnea^++^	279.82 ± 19.86	271.09 ± 17.64	286.17 ± 41.61	0.23	0.793
Insomnia^++^	293.03 ± 21.27	287.55 ± 18.89	343.94 ± 44.56	0.93	0.394
Appetite loss^++^	304.63 ± 18.83	296.67 ± 16.72	218.53 ± 39.45	2.53	0.081
Constipation^++^	325.42 ± 19.90	304.90 ± 17.67	292.33 ± 41.70	1.08	0.339
Diarrhea^++^	310.15 ± 19.83	326.64 ± 17.61	323.04 ± 41.55	0.64	0.530
Financial difficulties^++^	332.57 ± 15.95	340.29 ± 14.16	337.48 ± 33.41	0.22	0.806
THYCA-QoL					
Neuromuscular^++^	312.46 ± 21.37	279.97 ± 18.97	257.70 ± 44.77	2.394	0.092
Voice^++^	276.08 ± 18.95	286.05 ± 16.82	351.88 ± 39.69	1.953	0.143
Concentration^++^	297.85 ± 20.75	291.05 ± 18.42	262.67 ± 43.47	0.374	0.688
Sympathetic^++^	291.97 ± 21.65	295.72 ± 19.23	248.59 ± 45.37	0.621	0.538
Throat/mouth^++^	320.03 ± 21.63	305.31 ± 19.20	313.43 ± 45.31	0.431	0.650
Psychological^++^	298.05 ± 22.19	284.20 ± 19.71	252.20 ± 46.49	0.693	0.501
Sensory^++^	294.53 ± 21.69	277.50 ± 19.26	290.44 ± 45.45	0.588	0.556
Scar^++^	313.08 ± 14.07	313.87 ± 12.49	312.88 ± 29.47	0.003	0.997
Chilly^++^	293.95 ± 19.85	296.11 ± 17.62	221.43 ± 41.58	1.830	0.161
Tingling hands/feet^++^	307.58 ± 15.08	309.03 ± 13.39	270.78 ± 31.59	0.831	0.436
Weight gain^++^	328.04 ± 18.62	305.49 ± 16.53	311.45 ± 39.01	1.350	0.260
Headaches^++^	309.38 ± 20.04	292.23 ± 17.80	304.21 ± 41.99	0.692	0.501
Less interest in sex^++^	292.74 ± 18.69	296.73 ± 16.59	284.52 ± 39.15	0.09	0.915

Data are estimated marginal means (mean ± SE) on the rank-transformed. Model adjusted for age, sex, BMI, sunlight exposure season, medical insurance, family history of cancers, place of residence, and education level. ^+^Higher scores indicate better functioning. ^++^Higher scores indicate more symptoms. ^*^Significant: *p* < 0.05.

### Correlation between serum vitamin D and HRQoL domains

**Figure 2:**
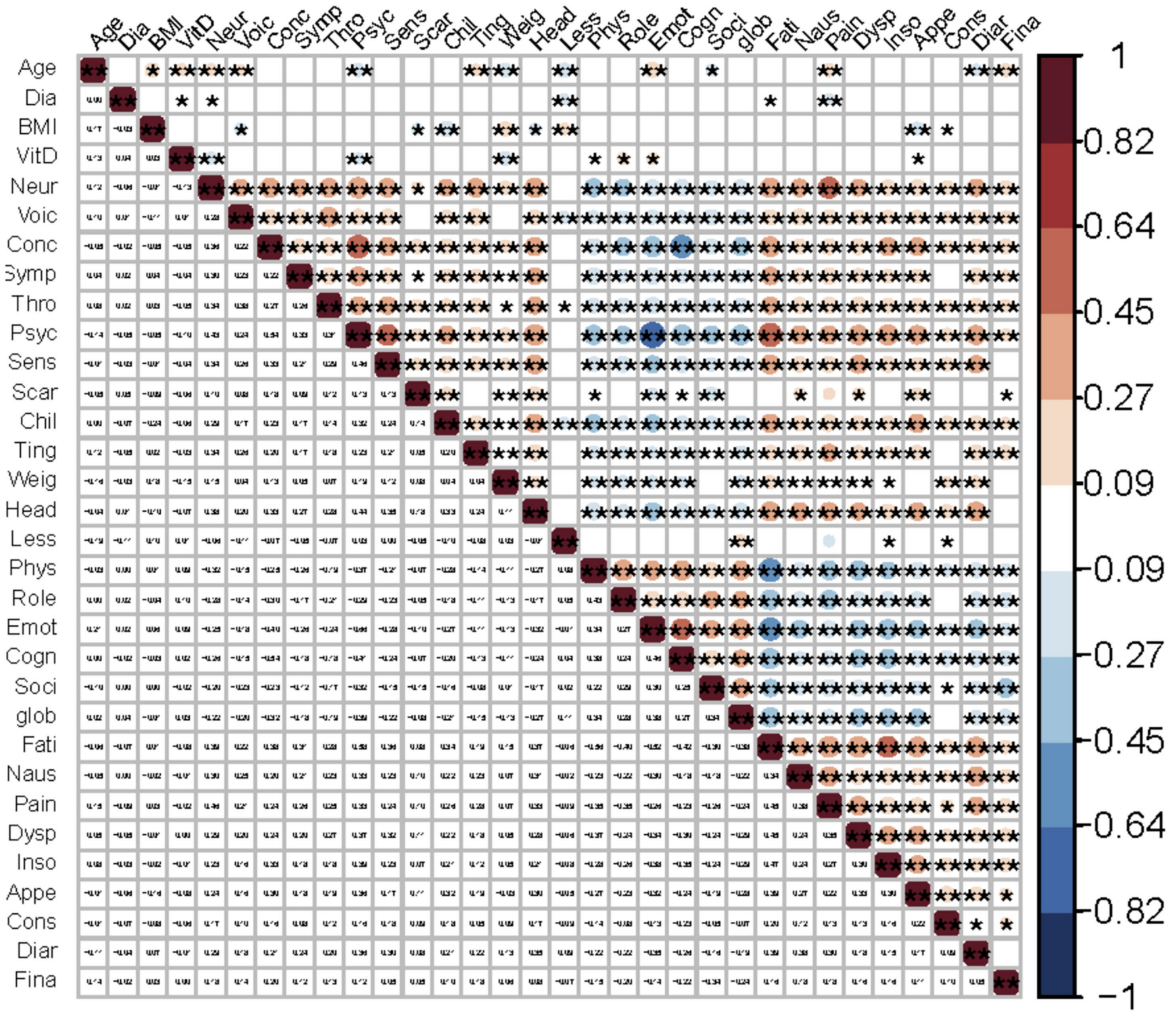
Heat map of correlations between serum vitamin D, age, BMI, maximum nodule diameter and QoL domains. Domains were abbreviated to the first four letters, such as physical functioning (Phys), emotional functioning (Emot), neuromuscular (Neur), etc. Red indicates positive correlations and blue indicates negative correlations. *p* < 0.01 is denoted by ** and *p* < 0.05 by *.

Spearman correlation analysis revealed that vitamin D levels showed positive correlations with physical (*ρ* = 0.087, *p* = 0.033), role (*ρ* = 0.105, *p* = 0.010), and emotional functioning (*ρ* = 0.095, *p* = 0.021) and negative correlations with symptoms of appetite loss (*ρ* = −0.081, *p* = 0.047), neuromuscular (*ρ* = −0.134, *p* = 0.001), psychological (*ρ* = −0.104, *p* = 0.011), and weight gain (*ρ* = −0.152, *p* < 0.001). Additionally, correlations between age, BMI, and maximum nodule diameter with the HRQoL scores also revealed several positive and negative associations, further emphasizing the complex interplay between these variables and HRQoL (Supplementary Table S2). The heatmap (Figure 2) visually illustrates these significant correlations, highlighting the strength and direction of the associations.

### Multivariable linear regression analyses

In multivariable linear regression, vitamin D was significantly associated with better physical and role functioning, as well as fewer symptoms of appetite loss, neuromuscular, psychological, and weight gain (all *p* < 0.05) after adjustment. No significant associations were observed in other HRQoL domains (Table 4). Detailed results of this analysis, which includes all variables tested for their relationship with vitamin D, are provided in Supplementary Table S3. These findings showed that vitamin D is independently associated with specific HRQoL domains in patients with PTC.

**Table 4:** Multivariable linear regression analysis of vitamin D levels and QoL scores.

QoL domains	Unstandardized coef.	95% CI	*p*	Standardized coef.
EORTC-QLQ-30				
Global health^+^	0.053	−0.053 to 0.158	0.330	0.042
Physical functioning^+^	0.094	0.021 to 0.166	0.011^*^	0.108
Role functioning^+^	0.086	0.005 to 0.167	0.038^*^	0.089
Emotion functioning^+^	0.102	−0.007 to 0.210	0.068	0.077
Cognitive functioning^+^	0.011	−0.093 to 0.115	0.837	0.009
Social functioning^+^	−0.044	−0.125 to 0.037	0.287	−0.045
Fatigue^++^	−0.072	−0.186 to 0.041	0.211	−0.053
Nausea/vomiting^++^	−0.016	−0.069 to 0.014	0.673	−0.018
Pain^++^	−0.041	−0.098 to 0.070	0.739	−0.014
Dyspnea^++^	0.015	−0.103 to 0.134	0.798	0.011
Insomnia^++^	0.022	−0.136 to 0.181	0.781	0.012
Appetite loss^++^	−0.133	−0.242 to −0.024	0.017^*^	−0.101
Constipation^++^	−0.018	−0.151 to 0.115	0.793	−0.011
Diarrhea^++^	−0.033	−0.150 to 0.084	0.578	−0.024
Financial difficulties^++^	−0.001	−0.088 to 0.087	0.984	−0.001
THYCA				
Neuromuscular^++^	−0.086	−0.153 to −0.019	0.012^*^	−0.105
Voice^++^	0.012	−0.066 to 0.090	0.763	0.013
Concentration^++^	−0.054	−0.141 to 0.032	0.218	−0.053
Sympathetic^++^	−0.039	−0.142 to 0.064	0.457	−0.032
Throat/mouth^++^	−0.043	−0.110 to 0.024	0.211	−0.054
Psychological^++^	−0.103	−0.191 to −0.014	0.023^*^	−0.097
Sensory^++^	−0.023	−0.116 to 0.070	0.627	−0.021
Scar^++^	−0.013	−0.107 to 0.082	0.792	−0.011
Chilly^++^	−0.101	−0.227 to 0.025	0.116	0.064
Tingling hands/feet^++^	−0.049	−0.132 to 0.033	0.241	−0.050
Weight gain^++^	−0.134	−0.250 to −0.018	0.024^*^	−0.093
Headache^++^	−0.059	−0.169 to 0.051	0.293	−0.045
Less interest in sex^++^	−0.015	−0.123 to 0.093	0.780	−0.011

Model adjusted for age, sex, BMI, sunlight exposure season, medical insurance, family history of cancers, place of residence, and education level. ^+^Higher scores indicate better functioning. ^++^Higher scores indicate more symptoms. ^*^Significant: *p* < 0.05.

### Multivariable logistic regression analyses

HRQoL domains that showed significant associations with vitamin D in the linear regression analysis were selected for logistic regression analysis with three models. In Model 3 (multivariable adjusted), higher vitamin D levels were independently associated with lower odds of impaired physical functioning (OR = 0.986, 95% CI 0.976–0.997, *p* = 0.016), role functioning (OR = 0.987, 95% CI 0.975–0.999, *p* = 0.035), symptoms of neuromuscular (OR = 0.985, 95% CI 0.974–0.995, *p* = 0.005), and weight gain (OR = 0.986, 95% CI 0.975–0.998, *p* = 0.018). For appetite loss and psychology, the associations were in the same (protective) direction but did not reach statistical significance in Model 3 (multivariable adjusted) (Table 5). Detailed results of this analysis, which includes all variables tested for their relationship with vitamin D, are provided in Supplementary Table S4.

**Table 5:** Logistic regression analysis of the association between vitamin D and QoL domains.

QoL domain	Model	OR	95% CI	*p*
Physical functioning^+^	Model 1 (unadjusted)	0.988	0.978 to 0.998	0.021^*^
	Model 2 (age and sex adjusted)	0.989	0.979 to 0.999	0.033^*^
	Model 3 (multivariable adjusted)	0.986	0.976 to 0.997	0.016^*^
Role functioning^+^	Model 1 (unadjusted)	0.987	0.976 to 0.998	0.026^*^
	Model 2 (age and sex adjusted)	0.986	0.975 to 0.998	0.018^*^
	Model 3 (multivariable adjusted)	0.987	0.975 to 0.999	0.035^*^
Appetite loss^++^	Model 1 (unadjusted)	0.991	0.981 to 1.001	0.067
	Model 2 (age and sex adjusted)	0.991	0.981 to 1.001	0.086
	Model 3 (multivariable adjusted)	0.992	0.981 to 1.002	0.122
Neuromuscular^++^	Model 1 (unadjusted)	0.985	0.975 to 0.995	0.003^*^
	Model 2 (age and sex adjusted)	0.984	0.974 to 1.035	0.002^*^
	Model 3 (multivariable adjusted)	0.985	0.974 to 0.995	0.005^*^
Psychology^++^	Model 1 (unadjusted)	0.990	0.980 to 1.001	0.063
	Model 2 (age and sex adjusted)	0.992	0.982 to 1.003	0.148
	Model 3 (multivariable adjusted)	0.994	0.982 to 1.005	0.264
Weight gain^++^	Model 1 (unadjusted)	0.982	0.972 to 0.992	0.001^*^
	Model 2 (age and sex adjusted)	0.985	0.974 to 0.995	0.004^*^
	Model 3 (multivariable adjusted)	0.986	0.975 to 0.998	0.018^*^

OR, odds ratio; CI, confidence interval. Model 1 was unadjusted. Model 2 was adjusted for age, sex. Model 3 was further adjusted for age, sex, BMI, sunlight exposure season, medical insurance, family history of cancers, place of residence, education level, maximum nodule diameter, and nodule focality. Vitamin D was analyzed as a continuous variable (per 1 nmol/L increase). ^+^Higher scores indicate better functioning. ^++^Higher scores indicate more symptoms. ^*^Significant: *p* < 0.05.

### Nomogram for HRQoL domain of weight gain

**Figure 3:**
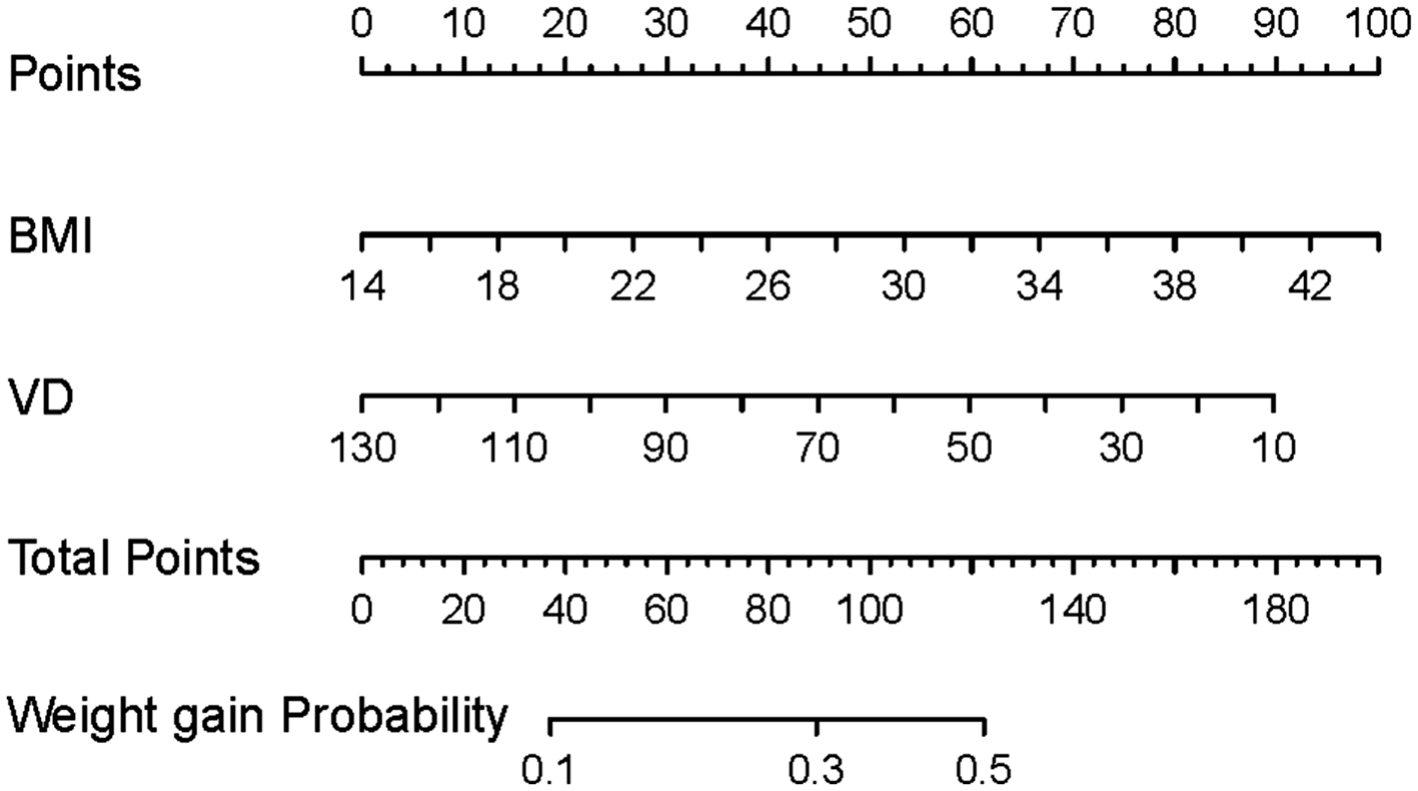
Nomogram for predicting the probability of QoL domain of weight gain in patients with PTC based on BMI and serum vitamin D levels. VD, serum vitamin D levels.

In linear and logistic regression analyses, both BMI and Vitamin D levels were independently associated with weight-gain domain. Based on these results, we conducted an exploratory nomogram to estimate the probability of weight gain symptoms. Higher BMI and lower Vitamin D levels correlated with an increased likelihood of weight gain. For example, individuals with a BMI of 30 and Vitamin D of 15 had a 50% probability of weight gain, while those with a BMI of 18 and vitamin D of 80 had a 20% probability (Figure 3).

### Nomogram performance and internal validation

**Figure 4:**
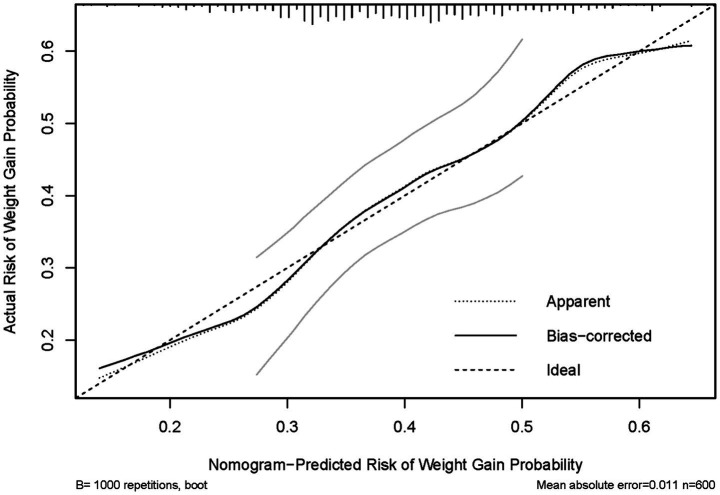
Calibration plot of the nomogram for predicting the risk of weight gain. The apparent and bootstrap-corrected calibration curves (1,000 resamples) are shown against the ideal 45° reference line.

The nomogram demonstrated fair discrimination, with a C-index of 0.628 (95% CI 0.583–0.673). Calibration of the nomogram showed good agreement between predicted and observed probabilities of weight gain. In the internal validation with 1,000 bootstrap resamples (*n* = 600), the bootstrap-corrected calibration curve was generally aligned with the ideal 45° reference line, with minor deviations at the lower and higher predicted risk ranges. The mean absolute error between predicted and observed probabilities was 0.011 (Figure 4).

## Discussion

The incidence of PTC has risen steadily in recent years. Despite its excellent prognosis, many patients experience persistent symptoms that impair HRQoL (23). Vitamin D, beyond its established role in calcium and bone metabolism, also affects immune function, cognition, and emotional health (24, 25). However, its impact on HRQoL in PTC patients remains largely unexplored. Therefore, we aimed to investigate the association between serum 25-hydroxyvitamin D levels and multiple HRQoL domains in patients with PTC.

Our results revealed a striking high prevalence of vitamin D deficiency and insufficiency, with 27.8 and 69.5% of participants. This finding underscores the potential relevance of vitamin D monitoring and optimization in this population. The results also revealed that patients with higher vitamin D levels (without exceeding the sufficiency threshold) had better physical and role functioning and fewer symptoms such as appetite loss, neuromuscular, psychological, and weight gain. Multivariable logistic regression analysis showed that higher vitamin D levels were independently associated with lower odds of impaired physical and role functioning, as well as reduced symptoms of neuromuscular and weight gain, suggesting that vitamin D status may be a potentially modifiable nutritional factor associated with specific aspects of HRQoL in PTC patients. These findings are consistent with evidence from other populations. In a cohort of older women, higher vitamin D intake was associated with better HRQoL, particularly in mental health domain (26). Similarly, in breast cancer patients, low vitamin D status has been linked to worse HRQoL outcomes including fatigue and depressive symptoms (16). Moreover, other studies reported that a strong association between higher vitamin D levels and improved physical and mental health, further supporting the multidimensional benefits of adequate vitamin D status (27, 28). Importantly, numerous studies have highlighted the alarmingly high prevalence of vitamin D deficiency, particularly among cancer patients (29, 30), underscoring the widespread nature of inadequate vitamin D status. Additionally, the nomogram combining BMI and serum vitamin D levels showed fair discrimination but good calibration in predicting weight gain symptom, suggesting its potential utility for risk stratification and individualized follow-up in PTC survivors. Previous research has similarly indicated the significant role of vitamin D in modulating weight gain (31).

Several biological mechanisms may explain the observed associations. First, vitamin D is essential for skeletal muscle metabolism and neuromuscular function (32, 33). Vitamin D deficiency disrupts calcium and muscle protein synthesis, leading to muscle weakness and fatigue (34), which is consistent with our findings that lower vitamin D levels were correlated with impaired physical functioning and more neuromuscular problems in patients with PTC. Improved muscle strength may translate into better role functioning by enabling patients to perform daily activities more effectively. Second, vitamin D acts as a neurosteroid hormone, with vitamin D receptors (VDRs) widely expressed in brain regions involved in emotional regulation, such as the prefrontal cortex, hippocampus, and amygdala, influencing neurotransmitter, neuroplasticity, neurotrophic factors, and hypothalamic-pituitary-adrenal (HPA) axis activity (35, 36). Dysregulation of these pathways is linked to psychological distress, providing a plausible explanation for the association between vitamin D and psychological well-being. However, social functioning can be influenced by many non-physical factors, such as anxiety and depression. Even with adequate vitamin D, the social functioning of PTC patients experiencing psychological distress may remain impaired.

Third, vitamin D plays a crucial role in energy homeostasis and adipose tissue regulation (37). VDRs are present in adipose tissue, and experimental evidence indicates that vitamin D deficiency promotes adipogenesis and fat storage (38). Low vitamin D has been associated with higher body fat percentage and impaired insulin sensitivity, further favoring fat accumulation (39). Finally, vitamin D influences appetite-regulating hormones such as leptin and exerts immunomodulatory and anti-inflammatory effects (40), suppressing pro-inflammatory cytokines (IL-1β, IL-6, TNF-*α*) that can reduce appetite via hypothalamic pathways (41). Taken together, these mechanisms provide biologically plausible pathways linking vitamin D to multiple HRQoL domains in patients with PTC and support the concept that vitamin D is a modifiable determinant of multidimensional HRQoL. Notably, these associations were domain-specific, as no significant effects were found in global health, fatigue, or cognitive functioning, suggesting that vitamin D may selectively influence particular HRQoL aspects in patients with PTC.

Previous studies have shown that multiple factors influence HRQoL in patients with PTC, including psychological distress such as anxiety and depression, which have also been implicated as risk factors for the development and progression of PTC (42, 43). In addition, different management strategies for PTC can markedly affect HRQoL. Active surveillance may increase psychological stress (44), whereas surgical resection can lead to persistent symptoms in long-term, thereby negatively affecting HRQoL (23). While our findings suggest an association between vitamin D status and certain HRQoL domains, vitamin D supplementation was not evaluated in this study. Future multicenter, longitudinal, and interventional studies are needed to confirm these findings and determine whether optimizing vitamin D status can improve HRQoL outcomes in patients with PTC.

This study has several strengths. It employed a prospective design with a relatively large sample size and used validated instruments (EORTC QLQ-C30 and THYCA-QoL) that enabled a comprehensive assessment of HRQoL for PTC patients. We also applied complementary statistical approaches, including rank-transformed GLM, correlation analysis, multivariable linear and logistic regression, and nomogram development with internal validation. These methods strengthen the robustness of our findings and help to partially mitigate the inherent subjectivity of HRQoL measures. Nonetheless, several limitations should be acknowledged. First, this was a single-center study, which may limit generalizability. Second, the present analysis was based on cross-sectional baseline data, preventing causal inferences. Finally, vitamin D supplementation was not evaluated. Future multicenter, longitudinal, and interventional studies are needed to confirm and determine whether vitamin D supplementation can causally improve HRQoL in PTC patients.

In conclusion, suboptimal vitamin D status was common among patients with PTC. Higher vitamin D levels were associated with better physical and role functioning and fewer neuromuscular, psychological, and weight gain symptoms. Assessment of vitamin D status may be considered as part of supportive care when clinically indicated. Randomized interventional studies are needed to determine whether optimizing vitamin D status can improve HRQoL outcomes in this population.

## Data Availability

The raw data supporting the conclusions of this article will be made available by the authors, without undue reservation.
