# Supplementary material for: Serum 25-hydroxyvitamin D and health-related quality of life in patients with papillary thyroid carcinoma: a prospective cohort study
Source: Front Nutr. 2026 Mar 10;13:1782902. doi: 10.3389/fnut.2026.1782902 (PMC13008716; doi:10.3389/fnut.2026.1782902)
Supplement: Supplementary file 1 [file Table_1.DOCX]

Supplementary Table S1. Pairwise comparisons of rank-transformed QoL scores across vitamin D status

| Comparison | Mean difference | Standard error | 95% CI | *P* |
| --- | --- | --- | --- | --- |
| EORTC-QLQ-30 |  |  |  |  |
| Global health + |  |  |  |  |
| Deficiency vs. Insufficiency | 5.412 | 16.50 | –34.21 to 45.04 | 1.000 |
| Deficiency vs Sufficiency | 19.32 | 45.53 | -89.99 to 128.62 | 1.000 |
| Insufficiency vs Sufficiency | 13.90 | 44.03 | -91.83 to 119.63 | 1.000 |
| Physical functioning + |  |  |  |  |
| Deficiency vs. Insufficiency | -34.23 | 16.05 | –72.75 to 4.30 | 0.1000 |
| Deficiency vs Sufficiency | -121.97 | 44.27 | -228.24 to -15.69 | 0.018 |
| Insufficiency vs Sufficiency | -87.74 | 42.82 | -190.532 to 15.06 | 0.123 |
| Role functioning + |  |  |  |  |
| Deficiency vs. Insufficiency | -20.88 | 13.09 | –52.31 to 10.54 | 0.333 |
| Deficiency vs Sufficiency | -37.52 | 36.11 | -124.20 to 49.17 | 0.898 |
| Insufficiency vs Sufficiency | -16.63 | 34.93 | -100.48 to 67.22 | 1.000 |
| Emotion functioning + |  |  |  |  |
| Deficiency vs. Insufficiency | -3.39 | 16.14 | –42.14 to 35.37 | 1.000 |
| Deficiency vs Sufficiency | -51.47 | 44.53 | -158.38 to 55.44 | 0.745 |
| Insufficiency vs Sufficiency | -48.08 | 43.07 | -151.49 to 55.33 | 0.794 |
| Cognitive functioning + |  |  |  |  |
| Deficiency vs. Insufficiency | -9.85 | 15.98 | –48.23 to 28.52 | 1.000 |
| Deficiency vs Sufficiency | -29.43 | 44.09 | -135.29 to 76.44 | 1.000 |
| Insufficiency vs Sufficiency | -19.58 | 42.65 | -121.97 to 82.82 | 1.000 |
| Social functioning + |  |  |  |  |
| Deficiency vs. Insufficiency | -4.45 | 12.87 | –35.34 to 26.44 | 1.000 |
| Deficiency vs Sufficiency | 118.60 | 35.49 | 33.39 to 203.81 | 0.003^*^ |
| Insufficiency vs Sufficiency | 123.05 | 34.33 | 40.63 to 205.47 | 0.001^*^ |
| Fatigue ++ |  |  |  |  |
| Deficiency vs. Insufficiency | 22.66 | 16.24 | –16.33 to 61.65 | 0.490 |
| Deficiency vs Sufficiency | 54.40 | 44.80 | -53.16 to 161.95 | 0.675 |
| Insufficiency vs Sufficiency | 31.73 | 43.33 | -72.30 to 135.77 | 1.000 |
| Nausea/vomiting ++ |  |  |  |  |
| Deficiency vs. Insufficiency | -14.96 | 13.78 | –48.05 to 18.13 | 0.835 |
| Deficiency vs Sufficiency | 35.02 | 38.02 | -56.27 to 126.31 | 1.000 |
| Insufficiency vs Sufficiency | 49.98 | 36.78 | -38.32 to 138.28 | 0.524 |
| Pain ++ |  |  |  |  |
| Deficiency vs. Insufficiency | -7.17 | 14.71 | –42.49 to 28.16 | 1.000 |
| Deficiency vs Sufficiency | 47.43 | 40.59 | -50.03 to 144.88 | 0.729 |
| Insufficiency vs Sufficiency | 54.59 | 39.26 | -39.67 to 148.85 | 0.495 |
| Dyspnea ++ |  |  |  |  |
| Deficiency vs. Insufficiency | 8.72 | 14.64 | –26.43 to 43.87 | 1.000 |
| Deficiency vs Sufficiency | -6.35 | 40.39 | -103.32 to 90.61 | 1.000 |
| Insufficiency vs Sufficiency | -15.07 | 39.07 | -108.86 to 78.32 | 1.000 |
| Insomnia ++ |  |  |  |  |
| Deficiency vs. Insufficiency | 5.48 | 15.68 | –32.16 to 43.13 | 1.000 |
| Deficiency vs Sufficiency | -50.91 | 43.25 | -154.75 to 52.93 | 0.719 |
| Insufficiency vs Sufficiency | -56.39 | 41.83 | -156.83 to 44.05 | 0.535 |
| appetite loss ++ |  |  |  |  |
| Deficiency vs. Insufficiency | 7.96 | 13.88 | –25.36 to 41.29 | 1.000 |
| Deficiency vs Sufficiency | 86.10 | 38.29 | -5.83 to 178.03 | 0.075 |
| Insufficiency vs Sufficiency | 78.14 | 37.04 | -10.78 to 167.06 | 0.106 |
| Constipation ++ |  |  |  |  |
| Deficiency vs. Insufficiency | 20.52 | 14.67 | –14.70 to 55.75 | 0.487 |
| Deficiency vs Sufficiency | 33.09 | 40.47 | -64.08 to 130.27 | 1.000 |
| Insufficiency vs Sufficiency | 12.57 | 39.15 | -81.42 to 106.56 | 1.000 |
| Diarrhea ++ |  |  |  |  |
| Deficiency vs. Insufficiency | -16.49 | 14.62 | –51.59 to 18.61 | 0.779 |
| Deficiency vs Sufficiency | -12.89 | 40.33 | -109.725 to 83.94 | 1.000 |
| Insufficiency vs Sufficiency | 3.60 | 39.01 | -90.06 to 97.26 | 1.000 |
| Financial difficulties ++ |  |  |  |  |
| Deficiency vs. Insufficiency | -7.72 | 11.76 | –35.94 to 20.50 | 1.000 |
| Deficiency vs Sufficiency | -4.91 | 32.43 | -82.77 to 72.95 | 1.000 |
| Insufficiency vs Sufficiency | 2.81 | 31.37 | -72.50 to 78.12 | 1.000 |
| THYCA |  |  |  |  |
| Neuromuscular ++ |  |  |  |  |
| Deficiency vs. Insufficiency | 32.49 | 15.75 | –5.33 to 70.31 | 0.119 |
| Deficiency vs Sufficiency | 54.76 | 43.45 | -49.56 to 159.08 | 0.624 |
| Insufficiency vs Sufficiency | 22.27 | 42.02 | -78.63 to 123.17 | 1.000 |
| Voice ++ |  |  |  |  |
| Deficiency vs. Insufficiency | -9.97 | 13.97 | –43.50 to 23.56 | 1.000 |
| Deficiency vs Sufficiency | -75.80 | 38.53 | -168.29 to -16.70 | 0.149 |
| Insufficiency vs Sufficiency | -65.83 | 37.26 | -155.294 to 23.64 | 0.233 |
| Concentration ++ |  |  |  |  |
| Deficiency vs. Insufficiency | 6.80 | 15.30 | –29.92 to 43.52 | 0.119 |
| Deficiency vs Sufficiency | 35.19 | 42.19 | -66.11 to -136.49 | 0.624 |
| Insufficiency vs Sufficiency | 28.39 | 40.81 | -69.60 to 126.37 | 1.000 |
| Sympathetic ++ |  |  |  |  |
| Deficiency vs. Insufficiency | -3.75 | 15.96 | –42.07 to 34.58 | 1.000 |
| Deficiency vs Sufficiency | 43.38 | 44.03 | -62.34 to 149.10 | 0.975 |
| Insufficiency vs Sufficiency | 47.13 | 42.59 | -55.13 to 149.39 | 0.807 |
| Throat/mouth ++ |  |  |  |  |
| Deficiency vs. Insufficiency | 14.72 | 15.96 | –23.56 to 52.99 | 1.000 |
| Deficiency vs Sufficiency | 6.60 | 44.03 | -98.99 to 112.18 | 1.000 |
| Insufficiency vs Sufficiency | -8.12 | 42.59 | -110.25 to 94.01 | 1.000 |
| Psychological ++ |  |  |  |  |
| Deficiency vs. Insufficiency | 13.85 | 16.36 | –25.43 to 53.13 | 1.000 |
| Deficiency vs Sufficiency | 45.85 | 45.13 | -62.50 to 154.20 | 0.930 |
| Insufficiency vs Sufficiency | 31.99 | 43.65 | -72.80 to 136.80 | 1.000 |
| Sensory ++ |  |  |  |  |
| Deficiency vs. Insufficiency | 17.03 | 15.99 | –21.36 to 55.42 | 0.862 |
| Deficiency vs Sufficiency | 4.10 | 44.11 | -101.81 to 110.00 | 1.000 |
| Insufficiency vs Sufficiency | -12.94 | 42.67 | -115.37 to 89.50 | 1.000 |
| Scar ++ |  |  |  |  |
| Deficiency vs. Insufficiency | -0.79 | 10.37 | –25.69 to 24.11 | 1.000 |
| Deficiency vs Sufficiency | 0.20 | 28.61 | -68.49 to 68.89 | 1.000 |
| Insufficiency vs Sufficiency | 0.99 | 27.67 | -65.45 to 67.43 | 1.000 |
| Chilly ++ |  |  |  |  |
| Deficiency vs. Insufficiency | -2.68 | 14.53 | –37.57 to 32.20 | 1.000 |
| Deficiency vs Sufficiency | 71.41 | 40.14 | -24.95 to 167.77 | 0.227 |
| Insufficiency vs Sufficiency | 74.09 | 38.87 | -19.23 to 167.41 | 0.171 |
| Tingling hands/feet ++ |  |  |  |  |
| Deficiency vs. Insufficiency | 1.09 | 11.14 | –25.67 to 27.84 | 1.000 |
| Deficiency vs Sufficiency | 42.75 | 30.78 | -31.15 to 116.66 | 0.496 |
| Insufficiency vs Sufficiency | 41.67 | 29.81 | -29.90 to 113.24 | 0.488 |
| Weight gain ++ |  |  |  |  |
| Deficiency vs. Insufficiency | 21.98 | 13.66 | –10.81 to 54.76 | 0.324 |
| Deficiency vs Sufficiency | 16.32 | 37.72 | -74.24 to 106.88 | 1.000 |
| Insufficiency vs Sufficiency | -5.66 | 36.53 | -93.36 to 82.05 | 1.000 |
| Headaches ++ |  |  |  |  |
| Deficiency vs. Insufficiency | 16.58 | 14.70 | –18.72 to 51.87 | 0.780 |
| Deficiency vs Sufficiency | 4.54 | 40.61 | -92.95 to 102.04 | 1.000 |
| Insufficiency vs Sufficiency | -12.03 | 39.33 | -106.44 to 82.38 | 1.000 |
| Less interest in sex ++ |  |  |  |  |
| Deficiency vs. Insufficiency | -4.00 | 13.78 | –37.07 to 29.08 | 1.000 |
| Deficiency vs Sufficiency | 8.22 | 38.00 | -83.02 to 99.46 | 1.000 |
| Insufficiency vs Sufficiency | 12.22 | 36.76 | -76.03 to 100.47 | 1.000 |

*Note: Pairwise comparisons were adjusted using Bonferroni correction; Dependent variable: rank-transformed scores; Model adjusted for age, sex, BMI, sunlight exposure season, medical insurance, family history of cancers, place of residence, and education level; +Higher scores indicate better functioning. ++Higher scores indicate more symptoms; ^*^ Significant: P < 0.05.*

**Supplementary Table S2**. Spearman correlation between serum vitamin D levels and QoL scores

| QoL domains | Vitamin D | | Age | | BMI | | Maximum nodule diameter | |
| --- | --- | --- | --- | --- | --- | --- | --- | --- |
|  | ρ | p-value | ρ | p-value | ρ | p-value | ρ | p-value |
| EORTC-QLQ-30 |  |  |  |  |  |  |  |  |
| global health + | 0.028 | 0.494 | 0.020 | 0.632 | -0.006 | 0.876 | 0.036 | 0.375 |
| Physical functioning + | 0.087 | 0.033^*^ | -0.029 | 0.478 | 0.012 | 0.772 | -0.001 | 0.989 |
| Role functioning + | 0.105 | 0.010^*^ | -0.005 | 0.903 | -0.044 | 0.278 | 0.021 | 0.600 |
| Emotion functioning + | 0.095 | 0.021^*^ | 0.210 | ＜0.001*^*^* | 0.061 | 0.139 | 0.023 | 0.575 |
| Cognitive functioning + | 0.019 | 0.649 | -0.004 | 0.929 | 0.027 | 0.504 | -0.024 | 0.560 |
| Social functioning + | -0.015 | 0.712 | -0.095 | 0.020*^*^* | -0.003 | 0.943 | -0.001 | 0.978 |
| Fatigue ++ | -0.075 | 0.066 | -0.057 | 0.164 | 0.005 | 0.897 | -0.068 | 0.097 |
| Nausea/vomiting ++ | -0.013 | 0.744 | -0.048 | 0.241 | -0.020 | 0.631 | -0.002 | 0.966 |
| Pain ++ | 0.023 | 0.568 | 0.147 | ＜0.001*^*^* | 0.032 | 0.432 | -0.093 | 0.023*^*^* |
| Dyspnea ++ | 0.000 | 0.992 | 0.053 | 0.193 | -0.011 | 0.789 | -0.051 | 0.216 |
| Insomnia ++ | -0.013 | 0.752 | 0.085 | 0.038*^*^* | -0.023 | 0.566 | -0.027 | 0.511 |
| Appetite loss ++ | -0.081 | 0.047^*^ | -0.013 | 0.756 | -0.157 | ＜0.001*^*^* | -0.064 | 0.115 |
| Constipation ++ | 0.059 | 0.151 | -0.011 | 0.786 | -0.076 | 0.062 | -0.074 | 0.071 |
| Diarrhea ++ | -0.007 | 0.870 | -0.113 | 0.006*^*^* | 0.073 | 0.075 | -0.036 | 0.380 |
| Financial difficulties ++ | -0.004 | 0.930 | 0.137 | 0.001*^*^* | 0.034 | 0.399 | -0.015 | 0.711 |
| THYCA-QoL |  |  |  |  |  |  |  |  |
| Neuromuscular ++ | -0.134 | 0.001^*^ | 0.124 | 0.002*^*^* | -0.008 | 0.840 | -0.057 | 0.165 |
| Voice ++ | 0.006 | 0.893 | 0.096 | 0.019*^*^* | -0.108 | 0.008*^*^* | 0.006 | 0.874 |
| Concentration ++ | -0.046 | 0.256 | -0.049 | 0.231 | -0.055 | 0.181 | -0.018 | 0.666 |
| Sympathetic ++ | -0.044 | 0.283 | 0.044 | 0.285 | 0.043 | 0.295 | 0.021 | 0.607 |
| Throat/mouth ++ | 0.053 | 0.193 | 0.077 | 0.060 | 0.027 | 0.506 | 0.023 | 0.571 |
| Psychological ++ | -0.104 | 0.011^*^ | -0.135 | 0.001*^*^* | -0.048 | 0.241 | -0.054 | 0.182 |
| Sensory ++ | -0.039 | 0.340 | -0.010 | 0.798 | -0.012 | 0.774 | -0.031 | 0.448 |
| Scar ++ | -0.058 | 0.160 | -0.047 | 0.255 | -0.092 | 0.025*^*^* | 0.053 | 0.193 |
| Chilly ++ | -0.060 | 0.140 | -0.003 | 0.944 | -0.235 | ＜0.001*^*^* | -0.074 | 0.070 |
| Tingling hands/feet ++ | -0.032 | 0.433 | 0.116 | 0.004 | 0.022 | 0.599 | -0.047 | 0.247 |
| Weight gain ++ | -0.152 | ＜0.001^*^ | -0.165 | ＜0.001*^*^* | 0.185 | ＜0.001*^*^* | -0.032 | 0.428 |
| Headaches ++ | -0.065 | 0.111 | -0.042 | 0.305 | -0.099 | 0.016*^*^* | 0.011 | 0.791 |
| Less interest in sex ++ | 0.007 | 0.871 | -0.193 | ＜0.001*^*^* | 0.105 | 0.010*^*^* | -0.106 | 0.009*^*^* |

*Notes: +Higher scores indicate better functioning; ++Higher scores indicate more symptoms; ^*^ Significant: P < 0.05.*

**Supplementary Table S3.** Multivariable linear regression analysis of factors associated with QoL in patients with PTC

| QoL domains | Variables | Unstandardized coef. | 95%CI | P | Standardized coef. |
| --- | --- | --- | --- | --- | --- |
| EORTC-QLQ-30 |  |  |  |  |  |
| Global health + | Vitamin D | 0.053 | -0.053 to 0.158 | 0.330 | 0.042 |
|  | Sex | -1.952 | -6.154 to 2.250 | 0.362 | -0.041 |
|  | Age | -0.011 | -0.181 to 0.159 | 0.901 | -0.006 |
|  | Sunlight exposure season | 0.822 | -2.773 to 4.416 | 0.654 | 0.019 |
|  | BMI | -0.010 | -0.592 to 0.393 | 0.691 | -0.018 |
|  | Place of residence | -0.362 | -5.988 to 5.263 | 0.899 | -0.006 |
|  | Family history of cancers | 1.530 | -2.789 to 5.848 | 0.487 | 0.029 |
|  | Medical insurance | -7.657 | -15.173 to -0.140 | 0.046^*^ | -0.084 |
|  | Education level | 0.896 | -1.948 to 3.741 | 0.536 | 0.029 |
| Physical functioning + | Vitamin D | 0.094 | 0.021 to -0.166 | 0.011^*^ | 0.108 |
|  | Sex | -3.467 | -6.345 to -0.589 | 0.018^*^ | -0.105 |
|  | Age | -0.018 | -0.134 to 0.099 | 0.763 | -0.014 |
|  | Sunlight exposure season | 1.507 | -0.955 to 3.968 | 0.230 | 0.051 |
|  | BMI | -0.071 | -0.409 to 0.266 | 0.677 | -0.018 |
|  | Place of residence | 0.651 | -3.202 to 4.503 | 0.740 | 0.014 |
|  | Family history of cancers | 1.243 | -1.714 to 4.201 | 0.409 | 0.034 |
|  | Medical insurance | 2.071 | -3.076 to 7.219 | 0.430 | 0.033 |
|  | Education level | 1.890 | -0.058 to 3.838 | 0.057 | 0.090 |
| Role functioning + | Vitamin D | 0.086 | 0.005 to 0.167 | 0.038^*^ | 0.089 |
|  | Sex | 1.749 | -1.467 to 4.966 | 0.286 | 0.048 |
|  | Age | -0.048 | -0.178 to 0.083 | 0.473 | -0.033 |
|  | Sunlight exposure season | -0.298 | -3.050 to 2.454 | 0.832 | -0.009 |
|  | BMI | -0.073 | -0.450 to 0.304 | 0.703 | -0.017 |
|  | Place of residence | 2.972 | -1.334 to 7.279 | 0.176 | 0.059 |
|  | Family history of cancers | 1.876 | -1.429 to 5.182 | 0.265 | 0.046 |
|  | Medical insurance | 2.275 | -3.479 to 8.029 | 0.438 | 0.032 |
|  | Education level | 1.533 | -0.645 to 3.710 | 0.167 | 0.066 |
| Emotion functioning + | Vitamin D | 0.102 | -0.007 to 0.210 | 0.068 | 0.077 |
|  | Sex | -2.363 | -6.686 to 1.959 | 0.283 | -0.047 |
|  | Age | 0.327 | 0.152 to 0.502 | ＜0.001^*^ | 0.166 |
|  | Sunlight exposure season | 0.160 | -3.538 to 3.858 | 0.932 | 0.004 |
|  | BMI | -0.009 | -0.516 to 0.497 | 0.971 | -0.002 |
|  | Place of residence | 3.388 | -2.399 to 9.175 | 0.251 | 0.049 |
|  | Family history of cancers | -2.800 | -7.242 to 1.642 | 0.216 | -0.050 |
|  | Medical insurance | 1.229 | -6.503 to 8.961 | 0.755 | 0.013 |
|  | Education level | -0.525 | -3.451 to 2.401 | 0.725 | -0.016 |
| Cognitive functioning + | Vitamin D | 0.011 | -0.093 to 0.115 | 0.837 | 0.009 |
|  | Sex | -1.090 | -5.224 to 3.044 | 0.605 | -0.023 |
|  | Age | 0.067 | -0.101 to 0.234 | 0.434 | 0.036 |
|  | Sunlight exposure season | -0.469 | -4.006 to 3.067 | 0.794 | -0.011 |
|  | BMI | -0.182 | -0.667 to 0.302 | 0.460 | -0.033 |
|  | Place of residence | -3.161 | -8.696 to 2.373 | 0.262 | -0.049 |
|  | Family history of cancers | -0.406 | -4.655 to 3.842 | 0.851 | -0.008 |
|  | Medical insurance | 5.991 | -1.404 to 13.386 | 0.112 | 0.067 |
|  | Education level | 2.639 | -0.159 to 5.437 | 0.064 | 0.088 |
| Social functioning + | Vitamin D | -0.044 | -0.125 to 0.037 | 0.287 | -0.045 |
|  | Sex | 3.311 | 0.086 to 6.536 | 0.044^*^ | 0.090 |
|  | Age | -0.121 | -0.252 to 0.009 | 0.068 | -0.084 |
|  | Sunlight exposure season | -1.876 | -4.635 to 0.883 | 0.182 | -0.057 |
|  | BMI | 0.278 | -0.100 to 0.655 | 0.150 | 0.064 |
|  | Place of residence | -2.590 | -6.908 to 1.727 | 0.239 | -0.051 |
|  | Family history of cancers | 1.182 | -2.132 to 4.497 | 0.484 | 0.029 |
|  | Medical insurance | -0.457 | -6.226 to 5.312 | 0.876 | -0.007 |
|  | Education level | 0.459 | -1.724 to 2.642 | 0.680 | 0.020 |
| Fatigue ++ | Vitamin D | -0.072 | -0.186 to 0.041 | 0.211 | -0.053 |
|  | Sex | 6.278 | 1.780 to 10.776 | 0.006^*^ | 0.122 |
|  | Age | -0.090 | -0.272 to 0.092 | 0.333 | -0.044 |
|  | Sunlight exposure season | 1.001 | -2.847 to 4.848 | 0.610 | 0.022 |
|  | BMI | 0.259 | -0.268 to 0.786 | 0.334 | 0.043 |
|  | Place of residence | -1.967 | -7.988 to 4.055 | 0.521 | -0.028 |
|  | Family history of cancers | 1.383 | -3.239 to 6.005 | 0.557 | 0.024 |
|  | Medical insurance | -4.163 | -12.208 to 3.883 | 0.310 | -0.042 |
|  | Education level | 0.526 | -2.519 to 3.570 | 0.735 | 0.016 |
| Nausea/ vomiting ++ | Vitamin D | -0.016 | -0.069 to -0.014 | 0.673 | -0.018 |
|  | Sex | 2.042 | -0.089 to 0.058 | 0.168 | 0.062 |
|  | Age | -0.033 | -0.867 to 4.950 | 0.579 | -0.026 |
|  | Sunlight exposure season | 0.204 | -2.284 to 2.692 | 0.872 | 0.007 |
|  | BMI | 0.079 | -0.261 to 0.420 | 0.648 | 0.020 |
|  | Place of residence | -0.168 | -4.062 to 3.725 | 0.932 | -0.004 |
|  | Family history of cancers | 0.009 | -2.979 to 2.998 | 0.995 | 0.000 |
|  | Medical insurance | 0.613 | -4.589 to 5.815 | 0.817 | 0.010 |
|  | Education level | 0.182 | -1.787 to 2.151 | 0.856 | 0.009 |
| Pain ++ | Vitamin D | -0.041 | -0.098 to -0.070 | 0.739 | -0.014 |
|  | Sex | 1.744 | -1.592 to 5.080 | 0.305 | 0.046 |
|  | Age | 0.221 | -0.086 to 0.356 | 0.001^*^ | 0.147 |
|  | Sunlight exposure season | 1.074 | -1.780 to 3.927 | 0.460 | 0.031 |
|  | BMI | 0.128 | -0.263 to 0.519 | 0.521 | 0.028 |
|  | Place of residence | -1.731 | -6.197 to 2.735 | 0.447 | -0.033 |
|  | Family history of cancers | 0.102 | -3.326 to 3.530 | 0.953 | 0.002 |
|  | Medical insurance | -1.453 | -7.420 to 4.514 | 0.633 | -0.020 |
|  | Education level | 0.334 | -1.924 to 2.592 | 0.771 | 0.014 |
| Dyspnea ++ | Vitamin D | 0.015 | -0.103 to 0.134 | 0.798 | 0.011 |
|  | Sex | 6.918 | 2.227 to 11.609 | 0.004^*^ | 0.129 |
|  | Age | 0.010 | -0.179 to 0.200 | 0.914 | 0.005 |
|  | Sunlight exposure season | 1.062 | -2.951 to 5.075 | 0.603 | 0.022 |
|  | BMI | 0.227 | -0.323 to 0.776 | 0.419 | 0.036 |
|  | Place of residence | -2.568 | -8.848 to 3.712 | 0.422 | -0.035 |
|  | Family history of cancers | -1.171 | -5.992 to 3.649 | 0.633 | -0.020 |
|  | Medical insurance | -2.514 | -10.904 to 5.877 | 0.557 | -0.025 |
|  | Education level | -2.881 | -6.056 to 0.294 | 0.075 | -0.084 |
| Insomnia ++ | Vitamin D | 0.022 | -0.136 to 0.181 | 0.781 | 0.012 |
|  | Sex | 9.547 | 3.251 to 15.842 | 0.003^*^ | 0.132 |
|  | Age | 0.345 | 0.090 to 0.599 | 0.008^*^ | 0.121 |
|  | Sunlight exposure season | 4.381 | -1.004 to 9.766 | 0.111 | 0.068 |
|  | BMI | 0.226 | -0.512 to 0.963 | 0.548 | 0.027 |
|  | Place of residence | -1.805 | -10.233 to 6.622 | 0.674 | -0.018 |
|  | Family history of cancers | -0.010 | -6.479 to 6.459 | 0.998 | 0.000 |
|  | Medical insurance | 6.711 | -4.549 to 17.972 | 0.242 | 0.049 |
|  | Education level | 4.260 | -0.001 to 8.521 | 0.050 | 0.093 |
| Appetite loss ++ | Vitamin D | -0.133 | -0.242 to -0.024 | 0.017^*^ | -0.101 |
|  | Sex | -1.279 | -5.598 to 3.040 | 0.561 | -0.026 |
|  | Age | -0.020 | -0.195 to 0.155 | 0.820 | -0.010 |
|  | Sunlight exposure season | 0.002 | -3.692 to 3.697 | 0.999 | 0.000 |
|  | BMI | -1.033 | -1.539 to -0.527 | ＜0.001^*^ | -0.176 |
|  | Place of residence | 2.533 | -3.249 to 8.315 | 0.390 | 0.037 |
|  | Family history of cancers | -0.416 | -4.855 to 4.022 | 0.854 | -0.007 |
|  | Medical insurance | -5.471 | -13.196 to 2.254 | 0.165 | -0.058 |
|  | Education level | -1.719 | -4.642 to 1.205 | 0.249 | -0.054 |
| Constipation ++ | Vitamin D | -0.018 | -0.151 to 0.115 | 0.793 | -0.011 |
|  | Sex | 9.660 | 4.377 to 14.943 | ＜0.001^*^ | 0.159 |
|  | Age | 0.047 | -0.167 to 0.261 | 0.666 | 0.020 |
|  | Sunlight exposure season | 2.906 | -1.613 to 7.425 | 0.207 | 0.053 |
|  | BMI | -0.220 | -0.839 to 0.399 | 0.485 | -0.031 |
|  | Place of residence | 7.017 | -0.055 to 14.089 | 0.052 | 0.084 |
|  | Family history of cancers | -3.013 | -8.442 to 2.415 | 0.276 | -0.044 |
|  | Medical insurance | 3.977 | -5.472 to 13.426 | 0.049^*^ | 0.034 |
|  | Education level | 0.571 | -3.004 to 4.147 | 0.754 | 0.015 |
| Diarrhea ++ | Vitamin D | -0.033 | -0.150 to 0.084 | 0.578 | -0.024 |
|  | Sex | -5.905 | -10.537 to -1.274 | 0.013^*^ | -0.111 |
|  | Age | -0.164 | -0.352 to 0.023 | 0.086 | -0.078 |
|  | Sunlight exposure season | 0.002 | -3.960 to 3.964 | 0.999 | 0.000 |
|  | BMI | 0.270 | -0.273 to 0.813 | 0.329 | 0.043 |
|  | Place of residence | 4.804 | -1.397 to 11.004 | 0.129 | -0.066 |
|  | Family history of cancers | 0.023 | -4.736 to 4.782 | 0.992 | 0.000 |
|  | Medical insurance | -0.494 | -8.778 to 7.791 | 0.907 | -0.005 |
|  | Education level | 2.582 | -0.553 to 5.717 | 0.106 | 0.076 |
| Financial difficulties ++ | Vitamin D | -0.001 | -0.088 to 0.087 | 0.984 | -0.001 |
|  | Sex | -1.673 | -5.143 to 1.798 | 0.344 | -0.041 |
|  | Age | 0.045 | -0.096 to 0.185 | 0.534 | 0.027 |
|  | Sunlight exposure season | 1.756 | -1.213 to 4.725 | 0.246 | 0.047 |
|  | BMI | -0.061 | -0.468 to 0.345 | 0.767 | -0.013 |
|  | Place of residence | 4.556 | -0.090 to 9.202 | 0.055 | 0.081 |
|  | Family history of cancers | 2.951 | -0.616 to 6.518 | 0.105 | 0.064 |
|  | Medical insurance | 5.794 | -0.415 to 12.002 | 0.067 | 0.074 |
|  | Education level | -6.331 | -8.680 to -3.981 | ＜0.001^*^ | -0.241 |
| THYCA |  |  |  |  |  |
| Neuromuscular ++ | Vitamin D | -0.086 | -0.153 to -0.019 | 0.012^*^ | -0.105 |
|  | Sex | 2.291 | -0.363 to 4.945 | 0.091 | 0.074 |
|  | Age | 0.167 | 0.060 to 0.275 | 0.002^*^ | 0.137 |
|  | Sunlight exposure season | 1.732 | -0.539 to 4.002 | 0.135 | 0.062 |
|  | BMI | 0.047 | -0.264 to 0.358 | 0.767 | 0.013 |
|  | Place of residence | -1.297 | -4.850 to 2.257 | 0.474 | -0.031 |
|  | Family history of cancers | -2.680 | -5.408 to 0.047 | 0.054 | -0.078 |
|  | Medical insurance | -3.437 | -8.184 to 1.311 | 0.156 | -0.058 |
|  | Education level | -1.025 | -2.822 to 0.771 | 0.263 | -0.052 |
| Voice ++ | Vitamin D | 0.012 | -0.066 to 0.090 | 0.763 | 0.013 |
|  | Sex | 0.320 | -2.774 to 3.415 | 0.839 | 0.009 |
|  | Age | 0.146 | 0.021 to 0.271 | 0.022^*^ | 0.105 |
|  | Sunlight exposure season | 0.059 | -2.588 to 2.706 | 0.965 | 0.002 |
|  | BMI | -0.475 | -0.837 to -0112 | 0.010^*^ | -0.114 |
|  | Place of residence | 0.453 | -3.690 to 4.595 | 0.830 | 0.009 |
|  | Family history of cancers | -0.551 | -3.731 to 2.629 | 0.734 | -0.014 |
|  | Medical insurance | -0.850 | -6.385 to 4.684 | 0.763 | -0.013 |
|  | Education level | -0.590 | -2.684 to 1.505 | 0.580 | -0.026 |
| Concentration ++ | Vitamin D | -0.054 | -0.141 to 0.032 | 0.218 | -0.053 |
|  | Sex | -0.856 | -4.298 to 2.586 | 0.625 | -0.022 |
|  | Age | -0.063 | -0.203 to 0.076 | 0.374 | -0.041 |
|  | Sunlight exposure season | 0.581 | -2.364 to 3.525 | 0.699 | 0.017 |
|  | BMI | -0.241 | -0.644 to 0.162 | 0.241 | -0.052 |
|  | Place of residence | 0.809 | -3.799 to 5.417 | 0.730 | 0.015 |
|  | Family history of cancers | -0.930 | -4.467 to 2.607 | 0.606 | -0.021 |
|  | Medical insurance | -5.259 | -11.415 to 0.898 | 0.094 | -0.070 |
|  | Education level | -1.607 | -3.936 to 0.723 | 0.176 | -0.064 |
| Sympathetic ++ | Vitamin D | -0.039 | -0.142 to -0.064 | 0.457 | -0.032 |
|  | Sex | 1.677 | -2.424 to 5.778 | 0.422 | 0.036 |
|  | Age | 0.048 | -0.118 to 0.214 | 0.571 | 0.026 |
|  | Sunlight exposure season | 0.850 | -2.658 to 4.358 | 0.634 | 0.020 |
|  | BMI | 0.412 | -0.068 to 0.893 | 0.093 | 0.075 |
|  | Place of residence | 6.364 | -0.874 to 11.854 | 0.023^*^ | 0.099 |
|  | Family history of cancers | -0.417 | -4.631 to 3.797 | 0.846 | -0.008 |
|  | Medical insurance | -6.111 | -13.446 to 1.225 | 0.102 | -0.068 |
|  | Education level | -0.513 | -3.289 to 2.263 | 0.717 | -0.017 |
| Throat/ mouth ++ | Vitamin D | -0.043 | -0.110 to 0.024 | 0.211 | -0.054 |
|  | Sex | 0.271 | -2.404 to 2.945 | 0.842 | 0.009 |
|  | Age | 0.057 | -0.052 to 0.165 | 0.304 | 0.047 |
|  | Sunlight exposure season | 0.281 | -2.007 to 2.569 | 0.810 | 0.010 |
|  | BMI | 0.043 | -0.270 to 0.357 | 0.787 | 0.012 |
|  | Place of residence | 2.127 | -1.454 to 5.707 | 0.244 | 0.051 |
|  | Family history of cancers | 0.013 | -2.735 to 2.762 | 0.993 | 0.000 |
|  | Medical insurance | -0.961 | -5.745 to 3.823 | 0.693 | -0.017 |
|  | Education level | -1.383 | -3.193 to 0.427 | 0.134 | -0.071 |
| Psychological ++ | Vitamin D | -0.103 | -0.191 to -0.014 | 0.023^*^ | -0.097 |
|  | Sex | 1.441 | -2.075 to 4.956 | 0.421 | 0.036 |
|  | Age | -0.142 | -0.284 to 0.001 | 0.051 | -0.090 |
|  | Sunlight exposure season | -1.068 | -4.075 to 1.940 | 0.486 | -0.030 |
|  | BMI | 0.060 | -0.352 to 0.472 | 0.774 | 0.013 |
|  | Place of residence | -1.320 | -6.025 to 3.386 | 0.582 | -0.024 |
|  | Family history of cancers | -0.056 | -3.669 to 3.556 | 0.976 | -0.001 |
|  | Medical insurance | -10119 | -7.407 to 5.169 | 0.727 | -0.015 |
|  | Education level | 0.878 | -1.501 to 3.258 | 0.469 | 0.034 |
| Sensory ++ | Vitamin D | -0.023 | -0.116 to 0.070 | 0.627 | -0.021 |
|  | Sex | 2.203 | -1.502 to 5.908 | 0.243 | -0.052 |
|  | Age | 0.049 | -0.101 to 0.199 | 0.522 | 0.030 |
|  | Sunlight exposure season | -0.724 | -3.893 to 2.446 | 0.654 | -0.019 |
|  | BMI | 0.062 | -0.373 to 0.496 | 0.781 | 0.012 |
|  | Place of residence | -3.556 | -8.516 to 1.404 | 0.160 | -0.062 |
|  | Family history of cancers | -2.071 | -5.878 to 1.736 | 0.286 | 0.044 |
|  | Medical insurance | -0.284 | -6.911 to 6.342 | 0.933 | -0.004 |
|  | Education level | 1.495 | -1.013 to 4.003 | 0.242 | 0.056 |
| Scar ++ | Vitamin D | -0.013 | -0.107 to 0.082 | 0.792 | -0.011 |
|  | Sex | 0.486 | -3.260 to 4.232 | 0.779 | 0.011 |
|  | Age | -0.093 | -0.244 to 0.059 | 0.229 | -0.055 |
|  | Sunlight exposure season | 2.504 | -0.700 to 5.708 | 0.125 | 0.065 |
|  | BMI | -0.488 | -0.927 to -0.049 | 0.030^*^ | -0.097 |
|  | Place of residence | 0.840 | -4.175 to 5.854 | 0.742 | 0.014 |
|  | Family history of cancers | -1.645 | -5.494 to 2.204 | 0.402 | -0.034 |
|  | Medical insurance | 1.454 | -5.246 to 8.154 | 0.670 | 0.018 |
|  | Education level | -1.830 | -4.365 to 0.706 | 0.157 | -0.067 |
| Chilly ++ | Vitamin D | -0.101 | -0.227 to 0.025 | 0.116 | 0.064 |
|  | Sex | 7.235 | 2.241 to 12.228 | 0.005^*^ | 2.543 |
|  | Age | 0.073 | -0.129 to 0.275 | 0.480 | 1.103 |
|  | Sunlight exposure season | -1.140 | -5.412 to 3.131 | 0.600 | 2.175 |
|  | BMI | -1.161 | -1.746 to -0.576 | ＜0.001^*^ | 0.298 |
|  | Place of residence | 1.359 | -5.327 to 8.044 | 0.690 | 3.404 |
|  | Family history of cancers | -0.418 | -5.550 to 4.714 | 0.873 | 2.613 |
|  | Medical insurance | 3.092 | -5.841 to 12.024 | 0.497 | 4.548 |
|  | Education level | -0.062 | -3.442 to 3.318 | 0.971 | 1.721 |
| Tingling hands/ feet ++ | Vitamin D | -0.049 | -0.132 to 0.033 | 0.241 | -0.050 |
|  | Sex | 1.418 | -1.851 to 4.688 | 0.395 | 0.038 |
|  | Age | 0.117 | -0.016 to 0.249 | 0.084 | 0.079 |
|  | Sunlight exposure season | -0.934 | -3.731 to 1.863 | 0.512 | -0.028 |
|  | BMI | 0.088 | -0.295 to 0.471 | 0.652 | 0.020 |
|  | Place of residence | -0.923 | -5.300 to 3.454 | 0.679 | -0.018 |
|  | Family history of cancers | -2.684 | -6.044 to 0.675 | 0.117 | -0.064 |
|  | Medical insurance | -3.037 | -8.885 to 2.810 | 0.308 | -0.042 |
|  | Education level | -2.519 | -4.731 to -0.306 | 0.026^*^ | -0.105 |
| Weight gain ++ | Vitamin D | -0.134 | -0.250 to -0.018 | 0.024^*^ | -0.093 |
|  | Sex | 7.619 | 3.000 to 12.238 | 0.001^*^ | 0.139 |
|  | Age | -0.348 | -0.535 to -0.161 | ＜0.001^*^ | -0.161 |
|  | Sunlight exposure season | 2.824 | -1.127 to 6.775 | 0.161 | 0.057 |
|  | BMI | 1.564 | 1.022 to 2.105 | ＜0.001^*^ | 0.242 |
|  | Place of residence | 4.249 | -1.935 to 10.432 | 0.178 | 0.057 |
|  | Family history of cancers | -2.542 | -7.289 to 2.204 | 0.293 | -0.041 |
|  | Medical insurance | 1.468 | -6.794 to 9.730 | 0.727 | 0.014 |
|  | Education level | 0.718 | -2.409 to 3.844 | 0.652 | 0.020 |
| Headache ++ | Vitamin D | -0.059 | -0.169 to 0.051 | 0.293 | -0.045 |
|  | Sex | 4.516 | 0.142 to 8.890 | 0.043 | 0.091 |
|  | Age | -0.074 | -0.251 to 0.103 | 0.414 | -0.037 |
|  | Sunlight exposure season | -1.605 | -5.347 to 2.137 | 0.400 | -0.036 |
|  | BMI | -0.295 | -0.808 to 0.218 | 0.259 | -0.050 |
|  | Place of residence | 4.709 | -1.146 to 10.564 | 0.115 | 0.069 |
|  | Family history of cancers | -2.600 | -7.094 to 1.895 | 0.256 | -0.047 |
|  | Medical insurance | -1.872 | -9.695 to 5.952 | 0.639 | -0.020 |
|  | Education level | -0.036 | -2.997 to 2.924 | 0.981 | -0.001 |
| Less interest in sex ++ | Vitamin D | -0.015 | -0.123 to 0.093 | 0.780 | -0.011 |
|  | Sex | -19.935 | -24.224 to -15.646 | ＜0.001^*^ | -0.371 |
|  | Age | -0.414 | -0.587 to -0.240 | ＜0.001^*^ | -0.195 |
|  | Sunlight exposure season | -1.059 | -4.728 to 2.610 | 0.571 | -0.022 |
|  | BMI | 0.044 | -0.458 to 0.547 | 0.862 | 0.007 |
|  | Place of residence | -3.721 | -9.463 to 2.021 | 0.204 | -0.051 |
|  | Family history of cancers | -0.103 | -4.511 to 4.304 | 0.963 | -0.002 |
|  | Medical insurance | -7.621 | -15.292 to 0.051 | 0.052 | 0.074 |
|  | Education level | 0.633 | -2.270 to 3.536 | 0.669 | 0.018 |

*Notes: Model adjusted for age, sex, BMI, sunlight exposure season, medical insurance, family history of cancers, place of residence, and education level; +Higher scores indicate better functioning. ++Higher scores indicate more symptoms; ^*^ Significant: P < 0.05.*

**Supplementary Table S4.** Logistic regression analysis of factors associated with QoL in patients with PTC

| QoL domains | Variables | OR | 95%CI | P |
| --- | --- | --- | --- | --- |
| Physical function |  |  |  |  |
|  | **Unadjusted** |  |  |  |
|  | Vitamin D | 0.988 | 0.978 to 0.998 | 0.021^*^ |
|  | BMI | 0.995 | 0.952 to 1.039 | 0.814 |
|  | Family history of cancers | 1.483 | 0.988 to 2.226 | 0.057 |
|  | Sunlight exposure season | 1.103 | 0.789 to 1.540 | 0.567 |
|  | Medical insurance | 1.281 | 0.615 to 2.668 | 0.508 |
|  | Place of residence | 0.921 | 0.556 to 1.525 | 0.749 |
|  | Maximum nodule diameter | 1.108 | 0.878 to 1.398 | 0.390 |
|  | Nodule Focality | 0.727 | 0.519 to 1.018 | 0.063 |
|  | Education level（ref=primary school） |  |  |  |
|  | Secondary/high school | 1.773 | 0.595 to 5.280 | 0.304 |
|  | College | 1.395 | 0.480 to 4.053 | 0.540 |
|  | Postgraduate or above | 0.923 | 0.295 to 2.888 | 0.891 |
|  | **Age and sex adjusted** |  |  |  |
|  | Vitamin D | 0.989 | 0.979 to 0.999 | 0.033^*^ |
|  | BMI | 1.020 | 0.973 to 1.070 | 0.407 |
|  | Family history of cancers | 1.470 | 0.975 to 2.215 | 0.066 |
|  | Sunlight exposure season | 1.123 | 0.801 to 1.574 | 0.501 |
|  | Medical insurance | 1.200 | 0.572 to 2.516 | 0.630 |
|  | Place of residence | 1.046 | 0.629 to 1.740 | 0.862 |
|  | Maximum nodule diameter | 1.084 | 0.858 to 1.370 | 0.499 |
|  | Nodule Focality | 0.736 | 0.522 to 1.037 | 0.080 |
|  | Education level（ref=primary school） |  |  |  |
|  | Secondary/high school | 1.857 | 0.619 to 5.568 | 0.269 |
|  | College | 1.429 | 0.475 to 4.296 | 0.525 |
|  | Postgraduate or above | 0.968 | 0.296 to 3.160 | 0.956 |
|  | **Multivariable adjusted** |  |  |  |
|  | Vitamin D | 0.986 | 0.976 to 0.997 | 0.016^*^ |
|  | BMI | 1.016 | 0.968 to 1.067 | 0.514 |
|  | Family history of cancers | 1.441 | 0.948 to 2.190 | 0.087 |
|  | Sunlight exposure season | 1.234 | 0.862 to 1.768 | 0.251 |
|  | Medical insurance | 1.125 | 0.518 to 2.443 | 0.766 |
|  | Place of residence | 0.996 | 0.573 to 1.730 | 0.988 |
|  | Maximum nodule diameter | 1.056 | 0.826 to 1.351 | 0.663 |
|  | Nodule Focality | 0.772 | 0.538 to 1.106 | 0.158 |
|  | Education level（ref=primary school） |  |  |  |
|  | Secondary/high school | 1.765 | 0.579 to 5.382 | 0.318 |
|  | College | 1.343 | 0.429 to 4.209 | 0.613 |
|  | Postgraduate or above | 0.971 | 0.286 to 3.302 | 0.963 |
| Role function |  |  |  |  |
|  | **Unadjusted** |  |  |  |
|  | Vitamin D | 0.987 | 0.976 to 0.998 | 0.026 |
|  | BMI | 1.017 | 0.971 to 1.066 | 0.468 |
|  | Family history of cancers | 1.000 | 0.639 to 1.565 | 1.000 |
|  | Sunlight exposure season | 0.832 | 0.581 to 1.191 | 0.315 |
|  | Medical insurance | 1.102 | 0.505 to 2.404 | 0.807 |
|  | Place of residence | 1.182 | 0.672 to 2.080 | 0.562 |
|  | Maximum nodule diameter | 0.931 | 0.716 to 1.211 | 0.594 |
|  | Nodule Focality | 0.922 | 0.641 to 1.326 | 0.662 |
|  | Education level（ref=primary school） |  |  |  |
|  | Secondary/high school | 0.740 | 0.258 to 2.124 | 0.576 |
|  | College | 0.690 | 0.248 to 1.920 | 0.478 |
|  | Postgraduate or above | 0.593 | 0.196 to 1.790 | 0.354 |
|  | **Age and sex adjusted** |  |  |  |
|  | Vitamin D | 0.986 | 0.975 to 0.998 | 0.018^*^ |
|  | BMI | 1.011 | 0.961 to 1.064 | 0.669 |
|  | Family history of cancers | 1.000 | 0.0638 to 1.569 | 0.999 |
|  | Sunlight exposure season | 0.822 | 0.573 to 1.180 | 0.288 |
|  | Medical insurance | 1.123 | 0.513 to 2.458 | 0.771 |
|  | Place of residence | 1.176 | 0.666 to 2.074 | 0.576 |
|  | Maximum nodule diameter | 0.935 | 0.718 to 1.218 | 0.618 |
|  | Nodule Focality | 0.920 | 0.636 to 1.331 | 0.658 |
|  | Education level（ref=primary school） |  |  |  |
|  | Secondary/high school | 0.716 | 0.248 to 2.065 | 0.536 |
|  | College | 0.649 | 0.225 to 1.874 | 0.424 |
|  | Postgraduate or above | 0.547 | 0.173 to 1.731 | 0.305 |
|  | **Multivariable adjusted** |  |  |  |
|  | Vitamin D | 0.987 | 0.975 to 0.999 | 0.035^*^ |
|  | BMI | 1.008 | 0.958 to 1.062 | 0.752 |
|  | Family history of cancers | 1.011 | 0.640 to 1.5495 | 0.964 |
|  | Sunlight exposure season | 0.913 | 0.623 to 1.338 | 0.641 |
|  | Medical insurance | 1.036 | 0457 to 2.347 | 0.933 |
|  | Place of residence | 1.334 | 0.720 to 2.470 | 0.359 |
|  | Maximum nodule diameter | 0.930 | 0.706 to 1.224 | 0.604 |
|  | Nodule Focality | 0.870 | 0.592 to 1.278 | 0.478 |
|  | Education level（ref=primary school） |  |  |  |
|  | Secondary/high school | 0.653 | 0.220 to 1.937 | 0.443 |
|  | College | 0.558 | 0.183 to 1.708 | 0.307 |
|  | Postgraduate or above | 0.496 | 0.149 to 1.655 | 0.254 |
| Appetite loss |  |  |  |  |
|  | **Unadjusted** |  |  |  |
|  | Vitamin D | 0.991 | 0.981 to 1.001 | 0.067 |
|  | BMI | 0.913 | 0.871 to 0.957 | ＜0.001^*^ |
|  | Family history of cancers | 1.074 | 0.710 to 1.625 | 0.734 |
|  | Sunlight exposure season | 0.865 | 0.619 to 1.208 | 0.395 |
|  | Medical insurance | 1.446 | 0.681 to 3.070 | 0.338 |
|  | Place of residence | 0.788 | 0.479 to 1.299 | 0.350 |
|  | Maximum nodule diameter | 0.904 | 0.709 to 1.154 | 0.419 |
|  | Nodule Focality | 0.941 | 0.671 to 1.320 | 0.726 |
|  | Education level（ref=primary school） |  |  |  |
|  | Secondary/high school | 0.848 | 0.306 to 2.352 | 0.752 |
|  | College | 0.873 | 0.324 to 2.351 | 0.789 |
|  | Postgraduate or above | 0.519 | 0.178 to 1.519 | 0.232 |
|  | **Age and sex adjusted** |  |  |  |
|  | Vitamin D | 0.991 | 0.981 to 1.001 | 0.086 |
|  | BMI | 0.914 | 0.869 to 0.962 | 0.001^*^ |
|  | Family history of cancers | 1.063 | 0.701 to 1.612 | 0.773 |
|  | Sunlight exposure season | 0.872 | 0.622 to 1.221 | 0.424 |
|  | Medical insurance | 1.385 | 0.650 to 2.952 | 0.399 |
|  | Place of residence | 0.807 | 0.488 to 1.333 | 0.403 |
|  | Maximum nodule diameter | 0.890 | 0.697 to 1.136 | 0.351 |
|  | Nodule Focality | 0.957 | 0.678 to 1.350 | 0.800 |
|  | Education level（ref=primary school） |  |  |  |
|  | Secondary/high school | 0.876 | 0.314 to 2.443 | 0.800 |
|  | College | 0.902 | 0.324 to 2.514 | 0.843 |
|  | Postgraduate or above | 0.546 | 0.179 to 1.666 | 0.288 |
|  | **Multivariable adjusted** |  |  |  |
|  | Vitamin D | 0.992 | 0.981 to 1.002 | 0.122 |
|  | BMI | 0.903 | 0.857 to 0.952 | ＜0.001^*^ |
|  | Family history of cancers | 1.090 | 0.710 to 1.675 | 0.692 |
|  | Sunlight exposure season | 0.933 | 0.651 to 1.335 | 0.703 |
|  | Medical insurance | 1.422 | 0.639 to 3.163 | 0.389 |
|  | Place of residence | 0.749 | 0.431 to 1.301 | 0.305 |
|  | Maximum nodule diameter | 0.875 | 0.679 to 1.129 | 0.304 |
|  | Nodule Focality | 0.945 | 0.657 to 1.360 | 0.762 |
|  | Education level（ref=primary school） |  |  |  |
|  | Secondary/high school | 0.917 | 0.319 to 2.637 | 0.872 |
|  | College | 0.958 | 0.324 to 2.831 | 0.938 |
|  | Postgraduate or above | 0.562 | 0.174 to 1.813 | 0.335 |
| Neuromuscular |  |  |  |  |
|  | **Unadjusted** |  |  |  |
|  | Vitamin D | 0.985 | 0.975 to 0.995 | 0.003^*^ |
|  | BMI | 0.976 | 0.935 to 1.018 | 0.258 |
|  | Family history of cancers | 1.400 | 0.937 to 2.091 | 0.100 |
|  | Sunlight exposure season | 0.859 | 0.621 to 1.188 | 0.357 |
|  | Medical insurance | 1.984 | 0.935 to 4.208 | 0.074 |
|  | Place of residence | 0.917 | 0.561 to 1.500 | 0.731 |
|  | Maximum nodule diameter | 0.830 | 0653 to 1.054 | 0.126 |
|  | Nodule Focality | 0.935 | 0.673 to 1.298 | 0.688 |
|  | Education level（ref=primary school） |  |  |  |
|  | Secondary/high school | 0.403 | 0.142 to 1.146 | 0.088 |
|  | College | 0.452 | 0.163 to 1.249 | 0.126 |
|  | Postgraduate or above | 0.273 | 0.092 to 0.809 | 0.019^*^ |
|  | **Age and sex adjusted** |  |  |  |
|  | Vitamin D | 0.984 | 0.974 to 1.035 | 0.002^*^ |
|  | BMI | 0.987 | 0.942 to 1.034 | 0.587 |
|  | Family history of cancers | 1.344 | 0.896 to 2.015 | 0.153 |
|  | Sunlight exposure season | 0.839 | 0.604 to 1.166 | 0.296 |
|  | Medical insurance | 1.844 | 0.863 to 3.938 | 0.114 |
|  | Place of residence | 0.984 | 0.598 to 1.619 | 0.950 |
|  | Maximum nodule diameter | 0.797 | 0.625 to 1.014 | 0.065 |
|  | Nodule Focality | 1.013 | 0.724 to 1.418 | 0.940 |
|  | Education level（ref=primary school） |  |  |  |
|  | Secondary/high school | 0.455 | 0.158 to 1.305 | 0.143 |
|  | College | 0.625 | 0.218 to 1.792 | 0.382 |
|  | Postgraduate or above | 0.402 | 0.130 to 1.241 | 0.113 |
|  | **Multivariable adjusted** |  |  |  |
|  | Vitamin D | 0.985 | 0.974 to 0.995 | 0.005^*^ |
|  | BMI | 0.978 | 0.932 to 1.026 | 0.361 |
|  | Family history of cancers | 1.370 | 0.904 to 2.076 | 0.138 |
|  | Sunlight exposure season | 0.974 | 0.685 to 1.384 | 0.974 |
|  | Medical insurance | 1.820 | 0.814 to 4.071 | 0.145 |
|  | Place of residence | 0.912 | 0.526 to 1.582 | 0.744 |
|  | Maximum nodule diameter | 0.791 | 0.614 to 1.019 | 0.070 |
|  | Nodule Focality | 0.930 | 0.652 to 1.327 | 0.688 |
|  | Education level（ref=primary school） |  |  |  |
|  | Secondary/high school | 0.411 | 0.137 to 1.228 | 0.111 |
|  | College | 0.543 | 0.177 to 1.667 | 0.286 |
|  | Postgraduate or above | 0.373 | 0.113 to 1.234 | 0.106 |
| Psychological |  |  |  |  |
|  | **Unadjusted** |  |  |  |
|  | Vitamin D | 0.990 | 0.980 to 1.001 | 0.063 |
|  | BMI | 0.957 | 0.913 to 1.002 | 0.064 |
|  | Family history of cancers | 1.049 | 0.684 to 1.609 | 0.826 |
|  | Sunlight exposure season | 0.825 | 0.585 to 1.166 | 0.276 |
|  | Medical insurance | 1.169 | 0.550 to 2.485 | 0.685 |
|  | Place of residence | 1.610 | 0.908 to 2.853 | 0.103 |
|  | Maximum nodule diameter | 0.727 | 0.549 to 0.962 | 0.025^*^ |
|  | Nodule Focality | 1.289 | 0.905 to 1.837 | 0.159 |
|  | Education level（ref=primary school） |  |  |  |
|  | Secondary/high school | 0.908 | 0.302 to 2.734 | 0.864 |
|  | College | 1.117 | 0.384 to 3.251 | 0.839 |
|  | Postgraduate or above | 1.527 | 0.495 to 4.709 | 0.461 |
|  | **Age and sex adjusted** |  |  |  |
|  | Vitamin D | 0.992 | 0.982 to 1.003 | 0.148 |
|  | BMI | 0.970 | 0.923 to 1.020 | 0.241 |
|  | Family history of cancers | 1.092 | 0.708 to 1.683 | 0.692 |
|  | Sunlight exposure season | 0.864 | 0.610 to 1.224 | 0.411 |
|  | Medical insurance | 1.182 | 0.551 to 2.534 | 0.667 |
|  | Place of residence | 1.571 | 0.881 to 2.800 | 0.126 |
|  | Maximum nodule diameter | 0.731 | 0.552 to 0.968 | 0.029^*^ |
|  | Nodule Focality | 1.215 | 0.8947 to 1.743 | 0.289 |
|  | Education level（ref=primary school） |  |  |  |
|  | Secondary/high school | 0.856 | 0.282 to 2.596 | 0.784 |
|  | College | 0.878 | 0.290 to 2.654 | 0.817 |
|  | Postgraduate or above | 1.184 | 0.366 to 3.825 | 0.778 |
|  | **Multivariable adjusted** |  |  |  |
|  | Vitamin D | 0.994 | 0.982 to 1.005 | 0.264 |
|  | BMI | 0.971 | 0.923 to 1.021 | 0.248 |
|  | Family history of cancers | 1.134 | 0.730 to 1.762 | 0.576 |
|  | Sunlight exposure season | 0.899 | 0.623 to 1.299 | 0.572 |
|  | Medical insurance | 1.164 | 0.524 to 2.587 | 0.710 |
|  | Place of residence | 1.613 | 0.868 to 2.998 | 0.130 |
|  | Maximum nodule diameter | 0.732 | 0.547 to 0.979 | 0.036 |
|  | Nodule Focality | 1.069 | 0.733 to 1.559 | 0.728 |
|  | Education level（ref=primary school） |  |  |  |
|  | Secondary/high school | 0.738 | 0.235 to 2.319 | 0.603 |
|  | College | 0.649 | 0.202 to 2.087 | 0.468 |
|  | Postgraduate or above | 0.874 | 0.255 to 2.996 | 0.830 |
| Weight gain |  |  |  |  |
|  | **Unadjusted** |  |  |  |
|  | Vitamin D | 0.982 | 0.972 to 0.992 | 0.001^*^ |
|  | BMI | 1.086 | 1.039 to 1.135 | ＜0.001^*^ |
|  | Family history of cancers | 1.018 | 0.675 to 1.533 | 0.933 |
|  | Sunlight exposure season | 0.640 | 0.460 to 0.891 | 0.008^*^ |
|  | Medical insurance | 0.655 | 0.331 to 1.299 | 0.226 |
|  | Place of residence | 0.653 | 0.400 to 1.069 | 0.090 |
|  | Maximum nodule diameter | 0.3933 | 0.735 to 1.183 | 0.565 |
|  | Nodule Focality | 1.210 | 0.865 to 1.692 | 0.266 |
|  | Education level（ref=primary school） |  |  |  |
|  | Secondary/high school | 0.917 | 0.321 to 2.619 | 0.871 |
|  | College | 1.331 | 0.481 to 3.682 | 0.582 |
|  | Postgraduate or above | 1.061 | 0.359 to 3.135 | 0.914 |
|  | **Age and sex adjusted** |  |  |  |
|  | Vitamin D | 0.985 | 0.974 to 0.995 | 0.004^*^ |
|  | BMI | 1.135 | 1.079 to 1.195 | ＜0.001^*^ |
|  | Family history of cancers | 1.092 | 0.718 to 1.661 | 0.680 |
|  | Sunlight exposure season | 0.677 | 0.483 to 0.947 | 0.023^*^ |
|  | Medical insurance | 0.670 | 0.333 to 1.350 | 0.263 |
|  | Place of residence | 0.591 | 0.356 to 0.981 | 0.042^*^ |
|  | Maximum nodule diameter | 0.952 | 0.747 to 1.213 | 0.690 |
|  | Nodule Focality | 1.083 | 0.767 to 1.530 | 0.649 |
|  | Education level（ref=primary school） |  |  |  |
|  | Secondary/high school | 0.787 | 0.272 to 2.278 | 0.658 |
|  | College | 0.805 | 0.279 to 2.325 | 0.689 |
|  | Postgraduate or above | 0.603 | 0.194 to 1.878 | 0.383 |
|  | **Multivariable adjusted** |  |  |  |
|  | Vitamin D | 0.986 | 0.975 to 0.998 | 0.018^*^ |
|  | BMI | 1.132 | 1.075 to 1.191 | ＜0.001^*^ |
|  | Family history of cancers | 1.090 | 0.701 to 1.693 | 0.703 |
|  | Sunlight exposure season | 0.762 | 0.531 to 1.093 | 0.139 |
|  | Medical insurance | 0.608 | 0.288 to 1.283 | 0.191 |
|  | Place of residence | 0.624 | 0.355 to 1.099 | 0.103 |
|  | Maximum nodule diameter | 1.025 | 0.790 to 1.330 | 0.850 |
|  | Nodule Focality | 1.018 | 0.702 to 1.475 | 0.925 |
|  | Education level（ref=primary school） |  |  |  |
|  | Secondary/high school | 0.940 | 0.308 to 2.867 | 0.913 |
|  | College | 1.103 | 0.351 to 3.468 | 0.867 |
|  | Postgraduate or above | 0.919 | 0.271 to 3.108 | 0.891 |

*Note: OR = odds ratio; CI = confidence interval. Model 1 was unadjusted；model 2 adjusted for age, sex; Model 3 was further adjusted for age, sex, BMI, sunlight exposure season, medical insurance, family history of cancers, place of residence, education level, maximum nodule diameter, and nodule Focality; Vitamin D was analyzed as a continuous variable (per 1 nmol/L increase); +Higher scores indicate better functioning. ++Higher scores indicate more symptoms; ^*^ Significant: P < 0.05.*
